# Deciphering *In Vitro* and *In Vivo* Pharmacological Properties of Seed and Fruit Extracts of *Flacourtia jangomas* (Lour.) Raeusch

**DOI:** 10.1155/2024/4035987

**Published:** 2024-07-26

**Authors:** Farhana Alam Ripa, Fowzia Alam, Fahmida Haque Riya, Yesmin Begum, Sharmin Akter Eti, Nusratun Nahar, Zebunnesa Ahmed, Sabrina Sharmin

**Affiliations:** ^1^ School of Pharmacy BRAC University, 41-Pacific Tower, Mohakhali, Dhaka 1212, Bangladesh; ^2^ Department of Pharmacy Southeast University, 251/A Tejgaon I/A, Dhaka 1208, Bangladesh; ^3^ United Hospital, Gulshan-2, Dhaka 1212, Bangladesh

## Abstract

The objective of the study was to evaluate the pharmacological properties of the methanolic extract of *Flacourtia jangomas* (Lour.) Raeusch fruits (PFJM) and seeds (SFJM), along with their soluble fractions in ethyl acetate (fruit: PFJE; seed: SFJE) and chloroform (fruit: PFJC; seed: SFJC). Our phytochemical analysis of the examined extracts confirmed the presence of various therapeutically active phytoconstituents, including flavonoids, tannins, glycosides, and alkaloids. Employing the DPPH (2,2-diphenyl-1-picrylhydrazyl) radical quenching method, SFJC exhibited the highest antioxidative potential, with an IC_50_ of 48.84, compared to ascorbic acid (IC_50_ 21.77). The thrombolytic activity was assessed through rapid clot analysis of human blood samples, revealing that SFJC demonstrated the highest thrombolytic activity (60.99 ± 2.28%) compared to streptokinase (72.89 ± 2.19%). In the protein denaturation antiarthritic test, the PFJE and SFJC extracts exhibited significant potency, achieving results of 74.28 ± 1.16% and 79.25 ± 0.83%, respectively, at a dose of 500 *μ*g/mL. All samples displayed notable anthelmintic activity by reducing *Pheretima posthuma* paralysis and death time in a dose-dependent manner compared to albendazole. In both *in vivo* analgesic tests, SFJC demonstrated substantial (*p* < 0.01) pain inhibition percentages (tail immersion: 49.46%; acetic acid writhing: 66.43%) at a dose of 600 mg/kg. During neuropharmacological screening, all extracts significantly (*p* < 0.01;  *p* < 0.05) and dose-dependently decreased the mice's locomotion activity and motor balance. In the thiopental-induced sedation assay, SFJC significantly decreased the sleep latency time (4.18 ± 0.24 min) and increased the duration of sleep time (85.20 ± 2.39 min) at a higher dose. All samples notably reduced blood glucose levels in the oral glucose tolerance test in a dose-responsive manner, and SFJC exhibited a considerable hypoglycemic impact (7.38 ± 0.44 mmoles/L at 600 mg/kg). The frequency of diarrheal episodes in mice during the antidiarrhea assessment was significantly decreased by the tested plant samples. These findings can serve as a reference for future endeavors to isolate pure bioactive compounds from this plant for the development of novel phytomedicines.

## 1. Introduction

Plants have a huge impact on human existence since they are the source of numerous, extremely effective alternative therapies for a range of human health hazards. The extraction of naturally occurring compounds with pharmacological properties is a crucial step in enhancing the value of plants that are traditionally used as food or medicine [1–5]. The research and exploration of medicinal plants for the development of novel therapeutic substances have been practiced since the dawn of time. Around four billion people, or 80% of the global population, are thought to reside in developing nations where herbal medicines are their main source of healthcare. In these communities, the use of herbs in traditional medicine is seen as an essential component of their culture [2, 3]. Since plant-based medicines are readily available and affordable, hundreds of different types of medicinal plants have been used for centuries in Bangladesh. The rural population has a long history of using those plants' therapeutic characteristics to meet their basic healthcare needs. However, these plants' continuing mystery has been their most alluring characteristic [2, 6]. We have selected the historically noteworthy medicinal plant *Flacourtia jangomas* (Lour.) Raeusch for further pharmacological investigation as part of our ongoing pharmacological research on Bangladeshi traditional medicinal plants.

The tropical fruit-bearing plant *F. jangomas*, also known as Paniala, Lukluki, Tokroi, or Painnagola, is a member of the Salicaceae family and is most frequently seen growing in the wild in Bangladesh. It is a little deciduous tree that typically grows to a height of 6–10 m, but can even grow to 14 m. The fruits are edible, colorful, and best consumed fresh in the summer when they are fully mature. In India, Bangladesh, and Myanmar, different parts of the plant are used as traditional medicines to treat a range of conditions, including gastrointestinal issues, inflammation, fever, diabetes, microbiological infections, asthma, diarrhea, jaundice, liver difficulties, nausea, and biliousness [4, 7–9]. Researchers revealed ostruthin, limolin, jangomolide, anthocyanin, alkaloids, *β*-carotene, carbohydrate, flavonoids, phenols, tannins, terpenoids, and saponins in several parts of the *F. jangomas* plant [7–11]. Few research studies on the pharmacological characteristics of the *F. jangomas* plant have been performed using various *in vitro* and *in vivo* tests, even though its distinct portions contain a variety of significant bioactive chemicals [8–11]. These studies confirm the plant's usage in traditional medicine in different diseases such as inflammation, diarrhea, toothache, and diabetes [8–12]. This sparked our interest in identifying the phytochemical components and examining the *in vitro* (antiarthritic, thrombolytic, and anthelmintic) and *in vivo* (analgesic, sedative, antidiarrheal, and hypoglycemic) pharmacological properties of the methanolic extracts of fruits and seeds of the aforementioned plant along with their various solvent fractions. Utilizing an array of *in vivo* and *in vitro* methodologies, we determined the diverse pharmacological attributes of the examined plant extracts to assess their multifarious medicinal properties and identify novel resources for an extensive range of pharmaceuticals.

## 2. Materials and Methods

### 2.1. Collection, Identification, and Extraction of Plant Materials

Fresh ripe *F. jangomas* fruits were collected from Gazipur district, Bangladesh in April, 2023 and were identified by a taxonomist from the Bangladesh National Herbarium in Mirpur, Dhaka (Accession no. DCAB- 87043). After precise washing, the seeds were separated from the fruits. Following a week of drying in the shade, they were ground into a fine powder and kept in sealed containers in a dark, cold, and dry room until they were processed. 500 g of powdered materials were macerated for 14 days with random shaking and stirring in two liters of 95 percent methanol in separate glass jars. After two weeks, the entire mixtures were separately filtered using a clean cotton bed and Whatman filter paper no. 1. The filtrates were concentrated to dryness in a rotary evaporator at 40°C under decreased pressure. The dried methanolic fruit (PFJM) and seed (SFJM) extracts of *F. jangomas* were fractionated with chloroform (fruit: PFJC; seed: SFJC), and ethyl acetate (fruit: PFJCE; seed: SFJE) sequentially in accordance with the Kupchan solvent-solvent partitioning protocol to separate the components in extracts according to their polarity [13]. Both *in vivo* and *in vitro* pharmacological characteristics of these plant samples were investigated.

### 2.2. Chemicals

Beximco Pharmaceuticals Ltd. of Bangladesh kindly provided diazepam, morphine, metformin, diclofenac sodium, indomethacin, loperamide, streptokinase, thiopental sodium, and albendazole to us. We procured ascorbic acid, acetic acid, and methanol (95%) from Sigma-Aldrich (USA). All other reagents utilized in the experiments were of analytical grade.

### 2.3. Test Organisms and Experimental Animals

Young Swiss albino mice of both sexes (age: 4-5 weeks and weight: 25–30 g) were utilized to conduct the *in vivo* pharmacological tests. Animal Resources Division of the International Center for Diarrheal Disease Research, Bangladesh (ICDDR, B) supplied the rodents. Prior to the pharmacological experiments, the mice were kept in a typical laboratory setting (room temperature 25 ± 1°C, relative humidity of 56%–60%, and a 12-h light/12-h dark cycle) for one week to acclimatize to the environment and provided with free access to ICCDR, B-formulated rodent food and water. Animal research was carried out in accordance with ICDDR, B norms that were approved by Southeast University's Animal Ethics Committee (Dhaka, Bangladesh) (SEU/Pharm/CECR/111/2023). Indian earthworms (*Pheretima posthuma*) were collected from Bangabandhu Sheikh Mujibur Rahman Agricultural University, Bangladesh for anthelmintic experiments.

### 2.4. Acute Oral Toxicity Test

On normal, mature, and nonpregnant female Swiss albino mice, acute oral toxicity tests based on OECD 425 standards were carried out on the examined extracts at a single dose of 2 g/kg [14]. On the first day, six starved mice received a limited dose of 2000 mg/kg of extracts (different mice for each extract). The mice were then closely monitored for 4 h, every 30 min, looking for any indications of toxicity and symptoms of mortality during the first 24 h. The subsequent four mice were given an equivalent dose of the extracts in a sequential manner for each group based on the results of the initial mouse. The mice were then kept individually and monitored daily for two weeks for any behavioral, autonomic, neurologic, or physical abnormalities that would be signs of intoxication. This observation was made over the course of 4 h with a 30 min break.

### 2.5. Qualitative Analysis of the Phytochemicals

The presence of unique bioactive components in the studied extracts (PFJM, PFJE, PFJC, SFJM, SFJE, and SFJC) was examined using conventional methods [3, 15]. The presence of phytochemicals constituted in the extracts was visually observed based on the color intensity, precipitation, and height of foam formation compared to the control (without the crude extract). Visual examination of color or foaming was used to evaluate whether a certain phytochemical group was present or absent [16].

### 2.6. Quantitative Estimation of Phytochemicals

#### 2.6.1. Estimation of Flavonoid Content

The flavonoid content of the test extracts was ascertained using the standard protocol as follows [13]. In 250 mL beakers, the dried sample extracts (2.50 g) were combined with precisely 50 mL of 95% ethanol, capped, and allowed to stand at 25°C for 24 h. Subsequently, the residue was extracted three times with the same quantity of ethanol and filtered using Whatman filter paper no. 42 (125 mm) shortly after the supernatant was discarded. Each sample's filtrates were then transferred to a beaker and allowed to dry in a water bath. After cooling, the dried filtrate was weighed using a desiccator until a constant weight was achieved. Finally, the following formula was used to estimate the percentage of flavonoids:

(1) $$\begin{array}{c} \%\text{ of flavonoid content}=\left[\frac{\text{weight of flavonoid}}{\text{weight of sample}}\right]\times 100\%. \end{array}$$

#### 2.6.2. Estimation of Phenolic Content

With a few modest adjustments, a Folin–Ciocalteu's reagent microplate assay technique was used to determine the tested samples' phenolic content. Next, using a standard curve made of gallic acid, the amount of phenols in the extracts was calculated and expressed in gallic acid equivalent per gram of the extract [15].

#### 2.6.3. Estimation of Alkaloid Content

The alkaloid content was measured by using the following technique as outlined by Andargie et al. [15]. Accordingly, 2.50 g of powdered sample extract was soaked into ethanol with 200 mL of 10% acetic acid in a 250 mL beaker and allowed to settle for 4 h. Shortly after filtration, the sample extracts were then concentrated to one-quarter of its baseline volume in a water bath. After that, 15 drops of concentrated ammonium hydroxide were gradually added to the extracts until complete precipitation was noticed. After 3 h of precipitation, the filtered liquid was then discarded, and the remnants were rinsed thoroughly with 20 mL of 0.1 M ammonium hydroxide and filtered using Gem filter paper (12.5 cm). Then, the residue was concentrated in a hot oven set at 40°C, and the weight was estimated using an electronic balance. Subsequently, the content of alkaloids was calculated and articulated in percentages by using the following equation:

(2) $$\begin{array}{c} \%\text{ of }a\text{lkaloid content}=\left[\frac{\text{weight of alkaloid}}{\text{weight of sample}}\right]\times 100\%. \end{array}$$

#### 2.6.4. Estimation of Tannin Content

The tannin content in the dried sample extracts was determined by using the Andargie et al.'s [15] method. The dried extracts (1 g) were taken and diluted in 1 mL of 95% ethanol. Following dilution, 50 *μ*l of the sample solution was poured into 100 *μ*l of vanillic acid solution (4% w/v) with 50 *μ*l of concentrated HCl. The absorbance was then measured immediately at 500 nm, and the quantity of tannin was computed using a calibration curve made from catechin as a standard. The results were presented in catechin equivalent (mg) per gram of the dried extract.

#### 2.6.5. Estimation of Saponin Content

With some minor modifications, the method described by Ezeonu and C. M. Ejikeme [17] was used to determine the quantitative amount of saponin. A 250 cm^3^ conical flask containing 5 grammes of each sample was filled to the exact volume with 100 cm^3^ of 90% aqueous ethanol. The mixture was heated to 55°C over a 4-hour period while being constantly stirred over a hot water bath. After filtering, the mixture's residue was again extracted using 100 cm^3^ of 95% aqueous ethanol, and it was heated for 4 hours at a steady 55°C while being constantly stirred. At 90°C, the combined extract evaporated to a volume of 40 cm^3^. After adding 20 cm^3^ of diethyl ether to the concentrate in a 250 cm^3^ separator funnel and agitating it strongly, the ether layer was discarded and the aqueous layer was recovered. There were two iterations of this cleansing procedure. After adding 60 cm^3^ of n-butanol, 10 cm^3^ of 5% sodium chloride was used for two extractions. The leftover solution was heated in a water bath for thirty minutes after the sodium chloride layer was discarded. It was then put into a crucible and dried in an oven to a consistent weight. A percentage was computed for the saponin content as follows:

(3) $$\begin{array}{c} \%\text{ of saponin content}=\left[\frac{\text{weight of saponin}}{\text{weight of sample}}\right]\times 100\%. \end{array}$$

#### 2.6.6. Estimation of Cyanogenic Glycoside

With some minor modifications, the method described by Ezeonu and C. M. Ejikeme [17] was used to determine the quantitative amount of cyanogenic glycoside. One gm of each powder sample was weighed and added to a 250 cm^3^ round-bottom flask, and 200 cm^3^ of distilled water was added. The sample was then left to stand for two hours to allow autolysis to take place. After adding an antifoaming agent (tannic acid), 20 cm^3^ of 2.5% NaOH (sodium hydroxide) was added to the sample and full distillation was performed in a 250 cm^3^ conical flask. The distillate was treated with 100 cm^3^ of cyanogenic glycoside, 8 cm^3^ of 6 M NH_4_OH (ammonium hydroxide), and 2 cm^3^ of 5% KI (potassium iodide). The mixture was then titrated with 0.02 M AgNO_3_ (silver nitrate) using a microburette on a black background. The persistent turbidity signifies the termination point. The content of cyanogenic glycoside in the sample was calculated as follows:

(4) $$\begin{array}{c} \text{cyanogenic glycoside}=\frac{\left(\text{titre value }\left(\text{cm}3\right)\times 1.08\times \text{extract volume}\right)}{\left(\text{aliquot volume }\left(\text{cm}3\right)\times \text{sample weight}\right)}\times 100. \end{array}$$

#### 2.6.7. Determination of Percentage Lipid

Each dry extract powder (2.50 g) was added to a thimble that was attached to a condenser and connected to a Soxhlet extractor chamber with a preweighed flat bottom. The flask was filled with 100 mL of petroleum ether (enough to induce reflux), and the wood sample's lipid was extracted for three hours by heating it on an electric hot plate set at 50°C. Petroleum ether was used as the extractant, and when the flask was cooled in a desiccator, the lipid was recovered. The lipid's value was determined by reweighing the flask and its contents [17]. Thus, the computed lipid content percentage was represented as follows:

(5) $$\begin{array}{c} \%\text{ of lipid conten}t=\left[\frac{\text{weight of lipid}}{\text{weight of sample}}\right]\times 100\%. \end{array}$$

#### 2.6.8. Determination of Carbohydrate

For three hours, 100 mg of the extract and 5 ml of 2.5 N HCl were hydrolyzed in a boiling water bath. After bringing it to room temperature, solid sodium carbonate was added and stirred until the effervescence subsided. After centrifuging the contents, distilled water was added to the supernatant to make 100 mL. This allowed for the pipetting of 0.2 mL of sample to make 1 mL of the volume using distilled water. Next, 1.0 mL of phenol reagent and 5.0 millilitres of sulfuric acid were added. The tubes were maintained for 20 minutes at 25–30°C. At 490 nm, the absorbance was measured [18].

#### 2.6.9. Determination of Protein

The dried samples were extracted by stirring with 50 ml of 95% ethanol (1 : 5 w/v) at 25°C for 24 h and centrifuged at 7,000 rpm for 10 min. 0.2 mL of the extract was pipetted out, and the volume was made to 1.0 ml with distilled water. 5.0 mL of alkaline copper reagent was added to all the tubes and allowed to stand for 10 min. Then, 0.5 mL of the Folin–Ciocalteu reagent was added and incubated in the dark for 30 min. The intensity of the color developed was read at 660 nm [18].

### 2.7. *In Vitro* Studies

#### 2.7.1. DPPH Radical Scavenging Screening for Antioxidant Activity of Extracts

With minimal modification, the previously published Blois DPPH scavenging assay [19–21] approach was used to assess the antioxidant capacity of the experimental plant extracts. To simply put, various concentrations of plant extracts (20–100 *μ*g/mL) were mixed with 3.0 mL of a methanol solution containing DPPH (40 *μ*g/mL). Next, using a UV spectrophotometer, the absorbance was determined at 517 nm. The formula used to calculate the free radical scavenging capacity is as follows:

(6) $$\begin{array}{c} \%\text{ of }i\text{nhibition}=\left[\frac{A_{\text{blank}}-A_{\text{sample}}}{A_{\text{blank}}}\times 100\%\right]. \end{array}$$

The absorbance for each group is represented here by A. The percent inhibition of DPPH scavenging versus the concentration of the test materials was then plotted on a graph to figure out the IC_50_ value (50 percent inhibition) for each tested sample.

#### 2.7.2. Study of Thrombolytic Activity

The thrombolytic test of extracts was conducted in accordance with a method that has been documented in the literature [16, 22] using streptokinase (SK) as a reference. Volunteer venous blood was divided into preweighted sterile Eppendorf tubes, which were then incubated at 37°C for 45 min. After the clot had formed, the serum was totally evacuated, and the clot's weight was determined. Each tube holding a clot had a unique addition of tested samples, SK (positive control), or an isotonic solution (negative control). The discharged fluid was collected after 90 min of incubation at 37°C, and tubes were reweighed. The weight variation represented as a percentage of clot lysis was calculated using the following equation:

(7) $$\begin{array}{c} \%\text{ clot lysis}=\left[\frac{\text{released clot weight}}{\text{clot weight}}\right]\times 100\%. \end{array}$$

#### 2.7.3. Study of Antiarthritic Activity by the Protein Denaturation Method

The antiarthritic effectiveness of plant extracts was evaluated following the prior approach with slight modification [16, 23]. The negative control solution only comprised of 0.45 mL of aqueous bovine serum albumin (BSA) solution and 0.05 mL of distilled water. While the positive control and test sample solutions contained 0.45 ml of BSA (5% aqueous solution) and 0.05 ml of distinct concentrations (100, 200, 300, 400, and 500 *μ*g/ml) of diclofenac sodium (reference drug) and tested extracts, respectively. All solutions' pH was attuned at 6.3 with 1N hydrochloric (HCl) acid. The samples had been warmed at 57°C for 3 min after being incubated for 30 min at 37°C and the absorbance was measured at 660 nm with an UV-visible spectrophotometer. The negative control simulates 100% protein denaturation. The findings were contrasted with those of sodium diclofenac. According to Ripa et al.'s approach, the % inhibition of protein denaturation was computed [16].

### 2.8. Membrane Stabilization Test

Plant extracts' anti-inflammatory efficacy is mostly determined by the HRBC membrane stabilization method [24]. An equivalent volume of sterilized Alsever's solution (2% dextrose, 0.8% sodium citrate, 0.5% citric acid, and 0.42% sodium chloride in water) was combined with the freshly drawn blood. The individuals in the sample were not given NSAIDs for two weeks prior to blood collection. Following a subsequent centrifugation of the collected blood for ten minutes at 3000 rpm, the pellet (packed cells) was rinsed three times with isosaline (0.85%; pH 7.2), and at last, a 10% (v/v) solution was prepared using isosaline. One mL of phosphate buffer (0.15 M; pH 7.4), two mL of hyposaline (0.36%), and half a mL of HRBC suspension were added to the various plant extract concentrations. Extracts were not added to the preparation of the standard or control. Various concentrations of indomethacin (1000, 2000, 3000, 4000, and 5000 *μ*g/mL) were employed as the reference medication and contrasted with corresponding plant extract quantities. After 30 minutes of incubation at 370°C, the reaction mixtures were centrifuged for 10 minutes at 3000 rpm. An estimation of the supernatant's concentration was 560 nm. The percentage hemolysis was calculated using the following equation:

(8) $$\begin{array}{c} \%\;h\text{emolysis}=\left[\frac{\left({\text{OD}}_{\text{test}}-{\text{OD}}_{\text{control}}\right)}{{\text{OD}}_{\text{control}}}\right]\times 100\%. \end{array}$$

The percentage of HRBC membrane stabilization was calculated using the following equation:

(9) $$\begin{array}{c} \%\;p\text{rotection}=\left[100-\frac{{\text{OD}}_{\text{test}}-{\text{OD}}_{\text{control}}}{{\text{OD}}_{\text{control}}}\;\right]\times 100\%. \end{array}$$

#### 2.8.1. Anthelmintic Test

Due to their anatomical and biological similarities to the human intestinal roundworm (*Ascaris lumbricoides)*, Indian earthworms (*P. posthuma*) were utilized in the contemporary research to assess the anthelmintic effectiveness of the studied extracts [21]. With a few small adjustments, we followed previous standard protocols here [16, 23, 25–28]. The worms (roughly 5–7 cm long and 0.3–0.5 cm wide) were carefully cleansed with normal saline to eliminate filth, and they were occasionally housed in lab conditions to adapt them to the surroundings before being utilized in the study. For the bioassay, we used three different concentrations (25, 50, and 75 mg/mL) of all extracts to measure the paralytic time and death rate of the worms. As a positive control, we used albendazole, while normal saline was used as a negative control. Periods of paralysis (completely no movement except when the worms were intensely shaken) and death (no mobility even if hot water was applied) in the experimental worms were noted.

### 2.9. *In Vivo* Studies

#### 2.9.1. Animal Experimental Design

For *in vivo* investigations, experimental mice were divided into multiple groups, each consisting of five to six animals according to the standard protocols for various experiments (Table 1). The extracts were administered in three different doses (200, 400, and 600 mg/kg) and saline solution was administered via oral gavage.

*(1) Analgesic Activity*. The peripheral writhing triggered by acetic acid and central tail immersion pain tests were used to examine the analgesic effect of the examined extracts.

(1) Peripheral analgesic test (acetic acid-induced writhing method)
The acetic acid writhing test was used to assess the peripheral analgesic effect in accordance with prior investigations with a little modification [29–31]. Initially, diclofenac sodium (standard) and plant extract samples were given orally to the animal models. Each mouse received an intraperitoneal (i.p.) injection of 0.6% acetic acid at a dose of 10 mL/kg body weight 40 min after receiving all treatments to elicit writhing (abdominal constrictions). The animals were placed in inverted flasks individually five min after the acetic acid injection, and the abdominal contractions and bending of the hind limbs were cumulatively timed for 30 min. In order to determine the analgesic activity, the average number of writhes and the % inhibition of writhing were computed [29, 30] as follows:
  (10) $$\begin{array}{c} \%\text{ of inhibition of writhing}=\left[\frac{N_{\text{control}}-N_{\text{test}}}{N_{\text{control}}}\right]\times 100\%, \end{array}$$
  where *N* represents the average number of writhing for each group.
(2) Central analgesic test (tail immersion method)
The central analgesic efficiency of the examined extracts was gauged by the technique of Karthik et al. [31]. In this experiment, extracts were administered orally to the animals at doses of 200, 400, and 600 mg/kg body weight. Following treatment, each mouse's tail up to 5 cm long was carefully placed in an organ bath with a thermostat set to 55 ± 1°C. The test was conducted on the animals that removed their tails from hot water in less than five seconds. The rodents' pain reaction time, or PRT (the amount of time in seconds needed for a mouse to retract its tail), was computed at intervals of 0, 30, and 60 min after the administration of the treatments. The following formula was used to compute the percentage of time the tail was submerged in relation to reference (morphine):
  (11) $$\begin{array}{c} \%\text{thermal stimulus protection}=\left[\frac{T_{\text{test}}-T_{\text{control}}}{N_{\text{control}}}\right]\times 100\%. \end{array}$$

In this instance, *T*_test_ denotes the test group's reaction time to pain, while *T*_control_ denotes the control group's reaction time to pain.

*(2) Neuropharmacological Assessment of Plant Extracts.*

1. Sleep test using thiopental sodium
2. The sedative activity of the examined extracts was evaluated using the thiopental sodium-induced sleeping time assessment, as described by Haque et al. [2]. Thirty min after oral ingestion of plant samples and reference diazepam (1 mg/kg), each mouse was administered sodium thiopental (40 mg/kg, i.p.) to induce sleep. The latent time (the interval between the administration of sodium thiopentone and the loss of the adjustment reflex) and sleep length (the interval between the loss and recovery of the adjusted reflex) in rodents were recorded.
3. Rotarod test
4. The motor coordination and performance of rodents were evaluated by a rotarod test according to the previously described method [32]. Animals were initially placed for two min on a horizontal, 20 rpm spinning hardwood bar with a diameter of 3 cm. Mice that passed the test were selected for the final assay. The selected mice were equally divided into 20 groups (5 mice each). The rodents of each group were individually kept on the rotarod for 2 min after receiving different treatments (group I: 1% tween 80; group II: diazepam: 1 mg/kg; and group III to group XX examined extracts: 200, 400, and 600 mg/kg).

*(3) Assessment of Antihyperglycemic Activity by Oral Glucose Tolerance Test (OGTT)*. The oral glucose tolerance test (OGTT) was conducted according to Ayele et al.'s technique [33] on overnight fasted mice (18 h) with minimal variation. Mice of either sex were split into 20 groups (each group consisted of 5 mice) at random. Baseline blood glucose level (BGL) was measured (shortly before distributing each agent based on their grouping). Animals orally received 2.5 g/kg of glucose solution 30 min after the administration of each treatment. Later, blood glucose levels were assessed after 30, 60, and 120 min.

*(4) Assessment of Antidiarrheal Activity*. The antidiarrheal efficacy of the investigated extracts was assessed using castor oil-induced and magnesium sulfate diarrheal models and gastrointestinal transit tests.

(1) Castor oil-induced diarrheal test
The technique suggested by Andargie et al. was followed in this investigation with simple minor amendments [15]. One hundred fasted mice (for 18 h) were equally divided into 20 groups (5 mice per group) and orally treated as indicated in the grouping and dosing segment. Each animal received one mL of castor oil orally to cause diarrhea after one hour of therapy. The animals were then separately kept in a plastic cage with a clean white paper-lined background and observed for the next 4 h to test for diarrhea, which was characterized as sloppy (wet), unformed feces and compared to the negative control group. Outcomes for the control group were regarded as being 100%. Each group's performance was evaluated using the percent inhibition (%) of diarrhea. The percentage of defecation inhibition was estimated as follows [34]:
  (12) $$\begin{array}{c} \%\text{ inhibition of defecation}=\left[\frac{D_{\text{control}}-D_{\text{test}}}{D_{\text{control}}}\right]\times 100\%, \end{array}$$
  where D stands for the average number of episodes of defecation in each group.
(2) Magnesium sulfate-induced diarrheal test
With a few minor adjustments, the previously reported approach was used in this model to cause diarrhea in rodents [35]. Prior to the experiment, the animals fasted for 18 h while still having access to fresh water. One hundred mice were distributed into twenty groups of five arbitrarily. All groups received the dosage and care described earlier in the castor oil-induced diarrhea paradigm. Inhibition percentages for feces and diarrhea were used to represent the results.
(3) Gastrointestinal transit test
This procedure is used to ascertain how experimental extracts affected rodent's gastrointestinal transit [35]. The test animals fasted for eighteen hours, just consuming water instead of food. For the castor oil-induced diarrhea test, the mice that were chosen were split into 20 groups (*n* = 5). All of the animals were given 1 mL of the charcoal meal (10% charcoal suspension in 5% gum acacia) orally once again after 30 minutes. All animals were sacrificed 30 minutes after the charcoal meal was administered, and the length of the intestinal tract that the charcoal meal covered, from the pylorus to the caecum, was calculated and reported as a percentage of the total distance travelled.

### 2.10. Statistical Analysis

All experimental data were reported as mean ± SEM (standard error of the mean). The results were statistically analyzed using analysis of variance (one-way ANOVA) followed by Dunnett's post hoc test for multiple comparisons to compare outcomes between groups, with *p* < 0.05 considered as significant. All statistical analyses were carried out using the IBM SPSS statistical software for the social sciences, version 26 for Windows (SPSS Inc., Armonk, New York, USA).

## 3. Results

### 3.1. Acute Oral Toxicity Test

The LD_50_ value of the studied extracts was established as 1000 mg/kg when administered orally to mice based on the findings of acute toxicity experiments. In order to conduct *in vivo* studies, three dose levels were chosen: 200, 400, and 600 mg/kg, p.o. body weight, which correspond to 20, 40, and 60% of the LD_50_ value (1000 mg/kg, p.o.). At the chosen doses, mice showed no signs of toxicity, such as death, or symptoms such as diarrhea, tremors, or unconsciousness. The experimental mice's eyes, skin color, fur, or mucous membranes did not alter throughout the investigation.

### 3.2. Phytochemical Screening

#### 3.2.1. Qualitative Phytochemical Screening

**Figure 1:**
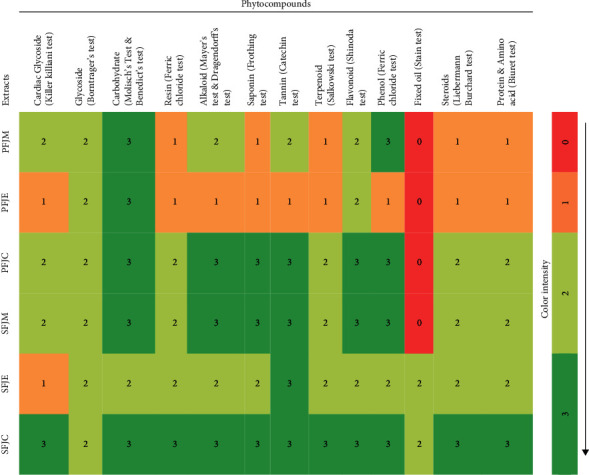
The heatmap shows the evaluation of phytochemical constituents in plant extracts. The red to green color indicates the amount from the absence (0 score) to the highest presence (3 score) of the phytochemicals in each sample. The color scale is shown on the right side of the heatmap.

Figure 1 displays the findings of the phytochemical screening of the examined extracts. Based on the color intensity, precipitation, and height of foam formation in comparison to the control (without the crude extract), the presence of phytochemicals included in the extracts was visually evaluated. The presence of plenty of valuable secondary metabolites was evident in the crude methanolic extracts of fruit and seeds, as well as in their fractions. Seed extracts were demonstrated to have a higher concentration of phytoconstituents than fruit extracts. All of the examined phytochemicals had been identified in seed extracts; however, fixed oil was not confirmed in the fruit extracts. Among all the biocomponents, carbohydrates were the most prevalent secondary metabolite, whereas fixed oil was found in the least amount in all the samples analyzed.

#### 3.2.2. Quantitative Determination of Phytochemical Constituents

The results of the quantitative analysis of different plant extracts are given in Table 2. From the table, we can observe that each experimented extract contains a number of valuable active bioactive components. Among all phytoconstituents, lipid was found in negligible amounts in all samples except SFJC (5.00 ± 0.74%).

### 3.3. *In Vitro* Studies

#### 3.3.1. DPPH Radical Scavenging Screening for Antioxidants

**Figure 2:**
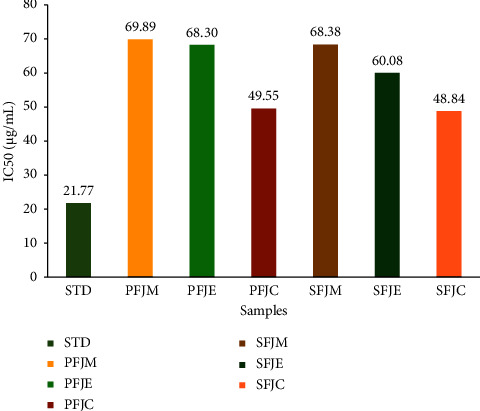
IC_50_ of the experimented extracts and standard. All experiments were performed in triplicate. Data are expressed as mean ± SD (*n* = 3; *p* < 0.05; *p* < 0.01) for all tested dosages.

The DPPH (2,2-diphenyl-1-picrylhydrazyl) radical scavenging technique was carried out to assess the antioxidant activity of *F. jangomas* fruit and seed extracts, and the results were represented as IC_50_ (Figure 2). All the examined extracts possess a concentration-dependent antiradical activity. Based on the computed IC_50_ values, the extracts exhibited medium DPPH scavenging activity with a range of 48.84–69.89 *μ*g/mL. In comparison to the reference ascorbic acid (IC_50_ = 21.77), the SFJC extract was found to be the most effective DPPH scavenger of all the extracts investigated (IC_50_ = 48.84). The free radical scavenging activity of the different extracts and ascorbic acid (STD) was in the following order: STD > SFJC > PFJC > SFJE > PFJE > SFJM > PFJM.

#### 3.3.2. Thrombolytic Efficacy of Plant Extracts

**Figure 3:**
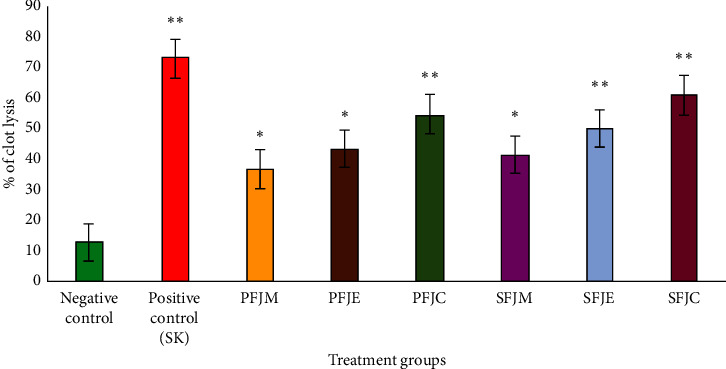
*In vitro* thrombolytic activity of *F. jangomas* extracts. Results are reported as the mean standard deviation of triplicate measurements (mean ± SEM; *n* = 3) and analyzed by one-way analysis of variance (ANOVA) trailed by Dunnett's test. ^*∗*^*p* < 0.05 and ^*∗∗*^*p* < 0.01 are significant compared to the negative control.

The crude methanolic fruit and seed extracts of *F. jangomas* and their different fractionates demonstrated notable thrombolytic activity (Figure 3). According to our study, SK exhibited the highest level of thrombolytic activity (72.89 ± 2.19% clot lysis; *p* < 0.01) in contrast to the negative control (12.63 ± 1.85%) after 90 min at 37°C temperature. SFJC exhibited the utmost degree of clot lysis among the investigated samples (60.99 ± 2.28%). In addition, the PFJC (54.56 ± 2.40%) extract showed considerable thrombolytic activity. PFJM (36.49 ± 2.79%) SFJM (41.21 ± 1.37%), and PFJE (43.18 ± 2.75%) showed a substantially reduced proportion of clot lysis even though the data were significant (*p* < 0.05) compared to the negative control.

#### 3.3.3. Protein Denaturation Antiarthritic Test

By employing a BSA (bovine serum albumin)-based antidenaturation assay, the evaluated extracts' antiarthritic activity was examined at five different concentrations (100–500 *μ*g/mL). The entire results are shown in Table 3. All plant extracts were found to suppress BSA denaturation in a concentration-dependent manner. In contrast to the standard drug (88.46 ± 0.73%), SFJC demonstrated a noticeably (*p* < 0.01) maximum percentage inhibition (79.25 ± 0.83%) at 500 *μ*g/mL. Furthermore, PFJC (74.28 ± 1.16%) and SFJE (71.72 ± 0.41%) also exhibited remarkable inhibition.

#### 3.3.4. Membrane Stabilizing Property

Table 4 displays the outcomes of the experiments for membrane stabilization. Comparison with the reference medication, indomethacin, revealed that every extract of *F. jangomas* showed concentration-dependent anti-inflammatory characteristics. The highest concentration examined was 5000 *μ*g/mL, and it was discovered that the extracts had anti-inflammatory properties in the following order: PFJC > PFJE > PFJM > SFJC > PFJE > PFJM. At the maximum dose, the reference drug indomethacin demonstrated 83% protection, whereas the SFJC extract demonstrated 77% protection.

#### 3.3.5. Anthelmintic Efficacy of Plant Extracts

Table 5 lists the results of the anthelmintic activity of the examined extracts in comparison to albendazole. All samples showed strong anthelmintic activity at various dosages, which led to the paralysis and death of earthworms in a dose-regulated way. At a higher concentration (75 mg/mL), SFJC was found to be the most efficient experimental extract for paralyzing (33.33 ± 1.52 min) and killing (40.67 ± 2.08 min) *P. posthuman*. Significant anthelmintic activity was also demonstrated by PFJC and SFJE extracts.

### 3.4. *In Vivo* Studies

#### 3.4.1. Peripheral and Central Analgesic Effectiveness of the Tested Extracts

The acetic acid-induced writhing method was used to test the extracts' ability to relieve peripheral pain, and all of the studied extracts significantly (*p* < 0.05; *p* < 0.01) decreased the number of writhes in mice when compared to the negative control at all doses. Diclofenac-Na recipients had the fewest writhing episodes (6.83 ± 1.47) when all treatment groups employing extracts were compared. Among all extracts, SFJC and PFJC exhibited the highest degree of inhibition (%) at a dose of 600 mg/kg, with comparable values of 66.43% and 62.56%. The outcomes are given in Table 6.

The tail immersion method was used to check the central analgesic properties of the studied samples. Like the acetic acid-induced writhing test, the investigated extracts also exhibited significant (*p* < 0.05;  *p* < 0.01) antinociceptive properties in a dose-dependent manner (Table 7). Reaction times for the negative control group did not alter significantly over time. The maximal analgesic activity for the standard group (morphine: 2 mg/kg) was noted at 30 min (*p* < 0.01), and its impact persisted for more than 90 min (*p* < 0.01). Compared to the negative control group, all analyzed sample groups showed notable reaction times at 60 min (*p* < 0.05;  *p* < 0.01), and their effects remained for more than 90 min (*p* < 0.01). Among the tested extracts, SFJC, SFJM, and PFJM showed potent analgesic properties at the utmost dose (600 mg/kg).

#### 3.4.2. Neuropharmacological Assessment of Plant Extracts

To assess the sedative potency and motor coordination of the examined extracts, the thiopental sodium-induced sleep time and rotarod tests were performed, respectively. In the thiopental-induced hypnosis test, the examined plant extracts showed dose-dependent (Table 8) slowing of the onset of sleep and an increase in sleep duration (*p* < 0.05;  *p* < 0.01). The extracts showed sleep-inducing effects akin to those of the reference medication diazepam. When compared to the negative control group, different extracts at varying doses significantly reduced the length of thiopental sodium-induced sleep and its latency in experimental mice.

According to the rotarod test outcomes, the extracts at a level of 200 mg/kg did not alter motor coordination (Table 9). However, compared to diazepam, which was more neurotoxic, extracts seem to be more neuroprotective since diazepam significantly lowered the mean latency of fall (28.2 ± 1.304 sec; *p* < 0.01).

#### 3.4.3. Oral Hypoglycemic Effect of the Examined Extracts

Table 10 provides a summary of how *F. jangomas* extracts affected oral glucose-loaded nondiabetic mice. Oral administration of the test samples caused mice in the glucose tolerance test to exhibit significantly more hypoglycemic activity (*p* < 0.05; *p* < 0.01) than the negative control group. There was no discernible difference between the initial fasting blood glucose levels (FBGLs) of any group when compared to the others. However, 30 min after the oral glucose challenge, all groups demonstrated a significant increase (*p* < 0.05; *p* < 0.01) in FBGL, demonstrating the induction of hyperglycemia. However, at the lowest dose (200 mg/kg), the investigated extracts were unable to produce observable changes over time. The uppermost activity in this investigation was shown by PFJC and SFJC extracts (at dosages of 400 and 600 mg/kg) and this activity persisted for 2 h after treatment (Table 10).

#### 3.4.4. Antidiarrheal Efficacy of the Investigated Extracts

All investigated extracts significantly (*p* < 0.05; *p* < 0.01, compared to group I) and dose-dependently reduced the overall number of diarrheal stools in the castor oil-induced diarrhea assessment (Table 11). SFJC demonstrated the most potent inhibitory activity out of all the extracts that were studied, and this activity changed with the dose. The study found that administering 200, 400, and 600 mg/kg of the SFJC extract, respectively, significantly reduced diarrhea to the levels of 40.29%, 64.18%, and 67.16%. Loperamide (2 mg/kg) demonstrated a more noticeable and significantly stronger (*p* < 0.01 compared to control) inhibitory effect on castor oil-induced fluid accumulation in comparison to the higher dose of SFJC (600 mg/kg).

The investigated extracts dose-relatedly decreased intestinal fluid secretion brought on by magnesium sulfate (Table 12). Compared to all extracts, the standard drug loperamide (2 mg/kg) produced a more pronounced and significantly greater (*p* < 0.01) inhibitory effect (80%) on fluid accumulation triggered by magnesium sulfate. In the same way as castor oil caused diarrhea, SFJC at 600 mg/kg dose considerably reduced (*p* < 0.01, compared to the negative control) the overall amount of diarrheal feces and showed the largest and most significant percentage of diarrheal inhibition (73.75%) among all tested extracts (Table 12).

In the case of the gastrointestinal motility test, we observed that all extracts significantly inhibited the gastrointestinal motility of charcoal meal in a dose-dependent manner as compared with the vehicle-treated group. The maximum effect was achieved by the PFJC extract at 600 mg/kg with the charcoal meal traversing 62.14% of the total length of the small intestine (Table 13). Besides the PFJC extract, the SFJC extract also showed promising inhibition at 400 and 600 mg/kg dose levels (57.56% and 59.11%, respectively).

## 4. Discussion

Phytochemicals have a substantial impact on the biological efficacy of medicinal plants. Secondary plant metabolites are currently used to make a significant number of medications. The development of novel plant-based medications is ongoing because herbal remedies are more affordable, low toxic, and associated with fewer health concerns than synthetic drugs. In addition, they are more readily available on the market. Most plant derivatives exhibit a variety of biological effects because of the presence of these bioactive components, such as analgesic, CNS depressant, hypoglycemic, antidiarrheal, antineoplastic, anticancer, anti-inflammatory, antioxidant, thrombolytic, and antiarthritis. Their presence also ensured their medicinal potential and therapeutic efficacy [1–7]. From ancient times, fruits and different plant parts of *F. jangomas* have been used to treat various diseases such as gastrointestinal issues, inflammation, fever, diabetes, microbiological infections, asthma, diarrhea, jaundice, and liver difficulties [8–12] but only few research studies were conducted on its pharmacological properties which provoked us to conduct the various *in vivo* (analgesic, sedative, hypoglycemic, and antidiarrheal) and *in vitro* (thrombolytic, anthelmintic, antiarthritic, and antioxidant) pharmacological characteristics of crude extracts of fruit and seed of *F. jangomas*. In the qualitative and quantitative phytochemical screening, the examined extracts showed the existence of copious valuable bioactive compounds such as carbohydrates, glycoside, resin, alkaloids, tannins, saponins, proteins, amino acids, flavonoids, fixed oil, and phenols (Figure 1 and Table 2) which supported the previous literature [6, 8–12]. Prior to conducting any *in vivo* research, acute oral toxicity testing is typically carried out since it can yield preliminary data regarding a substance's potential detrimental effects, establish an animal's dosage, and calculate the LD_50_ value of unknown extracts and phytochemicals [15, 35–37]. For this reason, we tested the investigated extract's acute oral toxicity in rodents. In the acute toxicity test, experimented extracts did not exhibit either mortalities or any abnormal alterations in general behavior, a noteworthy difference in weight allied with the treatment of extracts, or food/water intake as an indicator of serious poisoning in test rodents. Based on the approach of classifying acute toxicity, it may be inferred that the LD_50_ of the extracts is 1000 mg/kg, indicating that the extracts are typically thought to be harmless in acute consumption [35–37]. As a result, *in vivo* dosages of three high doses of the examined extracts (200, 400, and 600 mg/kg) were selected.

Free radicals weaken the body's resistance against disease and contribute to ageing, cancer, Alzheimer, atherosclerosis, angina pectoris, metabolic disorders, Parkinson's, complications from diabetes, rheumatoid arthritis, and other conditions [38, 39]. As a result of this, researchers are becoming more interested in creating plant-based natural antioxidants that can shield the body from the oxidative harm brought on by free radicals. The DPPH scavenging assay is the method that is most frequently used to evaluate the antioxidant capacity of plant materials. Their ability to donate hydrogen is thought to be what starts their antioxidant activity on DPPH [2, 40]. Despite having less activity than ascorbic acid (21.77 *μ*g/mL), our investigation was able to demonstrate that all extracts had a significant scavenging effect (IC_50_ values ranging from 69.89 to 48.84 *μ*g/mL; Figure 2) in a dose-dependent manner. A comparison of the antioxidant efficacies of the researched plant's seed and fruit extracts revealed that the chloroform fraction of the seed demonstrated good antioxidant property (IC_50_: 48.84 *μ*g/mL). Previous research proved that bioactive phytochemical components, particularly phenolic compounds (flavonoids, phenolic acids, and tannins), are crucial for both the antioxidant and free radical scavenging effects of plants (which were also identified in our tested extracts; Figure 1). The molecular patterns of polyphenols are also predictable; phenolic groups act as hydrogen donors and inhibit free radical oxidation. The extract's capacity to scavenge free radicals may be due to these polyphenolic chemicals [2, 38–40].

Numerous vascular issues are triggered by the formation of thrombi. Most of the thrombolytic drugs now on the market stimulate plasminogen, which in turn sparks the proteolytic breakdown of the cross-linked fibrin mesh by other enzymes. These drugs have severe side effects that are a significant clinical drawback and are connected to a number of restrictions, which prompted the need for an alternate treatment. Furthermore, epidemiologists' recent research using herbal and natural components has shown that natural thrombolytic/fibrinolytic drugs, as opposed to synthetic ones, lessen the incidence of thrombosis [22, 23, 41, 42]. In our investigation, crude methanolic fruit and seed extracts of *F. jangomas* and their fractions revealed notable thrombolytic activity in comparison to reference drug streptokinase (Figure 3). Among all extracts, SFJC exhibited the highest percentage of clot lysis (60.99 ± 2.28%) followed by PFJC (54.56 ± 2.40%). A number of investigations showed that tannins, flavonoids, alkaloids, and saponins are responsible for the clot-lysing activity. Researchers found that these phytochemicals prevent the formation of thrombus by inhibiting platelet aggregation, delaying the plasma recalcification time, and disrupting the fibrinogen and fibrin in a clot, resulting in fibrinolysis [34, 35] and the final dissolution of the clot. In our current study, we also confirmed the presence of the abovementioned bioactive constituents in qualitative and quantitative phytochemical screening tests, which supports the thrombolytic function of the studied extracts.

One of the main manifestations of arthritic disease is protein denaturation, which can produce autoantigens in numerous circumstances. Extrinsic stress, heat, organic solvents, strong acids, or bases can cause the loss of secondary and tertiary protein structures, a process known as protein denaturation [22–24]. Variations in electrostatic, hydrogen, hydrophobic, and disulfidec bonding are part of the denaturation mechanism [23, 43]. Comparing the extracts to the reference medication diclofenac sodium, the *in vitro* antiarthritic model protein denaturation experiment in this study showed a similar dose-dependent antiarthritic effect. At a dosage of 500 µg/mL, the chloroform fraction of *F. jangomas* seed (SFJC) displayed the maximum inhibition (79.25 ± 0.83%, Table 3). The increases in test sample absorbance compared to control showed that *F. jangomas* extracts can reduce the heat denaturation of protein (albumin). Lysosomes are essential to the inflammatory process because they release bactericidal enzymes, proteases, and activated neutrophils. In order to prevent inflammatory tissue damage, the release of these components must be regulated by stabilising the lysosomal membrane. The effect of any drug on RBC stabilization can be extrapolated to lysosomal membrane stabilization due to the similarities between RBC and lysosomal membranes [24]. Furthermore, RBC's strength depends on the integrity of their membranes, and membrane lysis occurs when RBCs come into contact with a hypotonic media [44]. Furthermore, damage to the lysosome membrane triggers the release of phospholipase A2 and lysosomal components, which in turn cause the breakdown of phospholipids to create inflammatory mediators [43]. Thus, another mechanism of the antiarthritic action is the suppression of RBC hemolysis in the hypotonic medium. Plant extracts and fractions demonstrated a tolerable dose-dependent stabilization of the RBC membrane in the current investigation (Table 4). The ability of *F. jangomas* to stabilize membranes may be related to its ability to obstruct the release of neutrophil lysosomal content. The potential protective effect on erythrocyte lysis could be recognized as a clear sign of the tested extracts' antiarthritic properties. According to the literature, plants with flavonoids and phenolic compounds have antiarthritic properties, and our phytochemical screening confirmed the presence of the aforementioned biomolecules in the analyzed extracts [22–24, 44, 45].

Worm or parasitic infections, one of the most common human infections, have a huge influence on a sizeable portion of the world's population. The advent of resistant strains, the discovery of anthelmintic medication residues in animal products, and the toxicity of synthetic pharmaceuticals have reignited interest in using natural remedies [46]. New physiologically active molecules that are compatible with human physiology and have no negative effects or less than those of synthetic chemicals can also be produced by natural resources such as plants [25–28, 45, 46]. In the anthelmintic investigation, we make a distinction between plant extracts and regular albendazole based on how long earthworms remain paralyzed until they die. We found a statistically significant correlation between extract-graded concentrations, exposure times, and adult parasite mortality in this instance (Table 5). The earthworms' time to paralysis or death was inversely correlated with the effectiveness of plant extracts. The chloroform fraction of seed showed possible reductions in paralysis (33.33 ± 1.52 min) and death (40.67 ± 2.08 min) periods compared to the reference drug (paralysis: 28.67 ± 1.15 min and death: 40.33 ± 1.52 min respectively) at a higher dose (75 mg/mL). Several studies revealed that the anthelmintic activity is caused by tannins, flavonoids, alkaloids, and phenolic compounds. Tannins have the capacity to attach to free proteins in the gastrointestinal system of an animal or to glycoprotein on the cuticle of a parasite, both of which can be lethal [46]. Saponins predominantly irritate the mucous membranes simultaneously, which leads to paralysis or possibly death in the end. Alkaloids may potentially have an immediate effect on helminths' neurological systems and preparasitic stages [27, 47]. Phenolic chemicals, alkaloids, flavonoids, tannins, and saponins were found in our phytochemical investigation. In light of this, we can consider that the seed and fruit extracts of *F. jangomas* are alternative potential sources for the development of novel anthelmintic drugs.

In order to assess the anticipated mechanism of action of the extracted ingredient, the antinociceptive properties of *F. jangomas* fruit and seed extracts were evaluated using the authentic peripheral (acetic acid-induced writhing) and central (tail immersion) techniques [29, 48]. The acetic acid-induced writhing test is a highly suggested model for screening the peripheral analgesic potentials of test compounds due to its sensitivity and capacity to identify antinociceptive effects of natural products and test compounds at dose levels that remain inactive for other methods [29, 31]. Pain sensation is represented by the writhing model of acetic acid induced through initiating locally inflaming response and hypothesized that peritoneum mast cells, acid-sensing ion channels, and prostaglandin paths mediate the response by releasing histamine, prostaglandins (PGs), bradykinin, serotonin, cyclooxygenase (COX), and cytokines [29, 48–56]. These inflammatory chemicals are produced by the COX pathway during the metabolism of arachidonic acid, and they cause chemosensitive nociceptors to become active, which in turn causes inflammatory pain [49, 50]. Recent studies showed that due to the injection of acetic acid, a huge amount of PGE2 and PGF2á was liberated and caused constriction of the muscle of the abdomen by the expansion of front paws and prolongation of the body (writhing) within the first 30 min. In addition, intraperitoneal acetic acid injections (painkillers) have been shown to promote vascular fluid permeability and vasodilation [48–56]. The number of abdominal writhes caused by acetic acid in mice was considerably decreased by oral administration of the plant extracts in the current investigation (*p* < 0.01;  *p* < 0.05) (Table 6). Therefore, the antinociceptive effects of PFJM, PFJE, PFJC, SFJM, SFJE, and SFJC may be attained by either preventing the release of endogenous nociceptive mediators or by preventing the penetration of both the blood-brain barrier and vascular fluid levels. Despite being an extremely sensitive pain test on animals, the acetic acid-induced writhing method is not a discriminating one. Therefore, the tail immersion test was also conducted, which represents the centrally acting analgesic action, to confirm that the plant exhibits analgesic activity [31, 48–51]. Tail immersion is involved in spinal reflexes that act through the opioid *μ*_2_ and *δ* receptors [48]. When compared to the negative control groups, we saw that all of the investigated extracts considerably (*p* < 0.01;  *p* < 0.05) sped up the mice's reaction times. The chloroform fractionate of *F. jangomas* fruit (PFJC) was found to be most effective (Table 7) and active for more than 90 min (5.00 ± 0.29 sec). By stimulating the periaqueductal grey matter (PAG), which releases endogenous peptides such as endorphin and enkephalin, the investigated plant extracts (PFJM, PFJE, PFJC, SFJM, SFJE, and SFJC) may have antinociceptive effects on the central nervous system. At the synaptic connection of the dorsal horn, these endogenous peptides travel down the spinal cord and prevent the transmission of pain impulses [50–55]. Based on the results of the two antinociceptive tests, it is possible that our molecules have both central and peripheral analgesic actions, similar to NSAIDs and opioids. It was previously reported in the literature that plants with substantial analgesic efficacy contained terpenoids, tannins, glycosides, alkaloids, saponins, phenols, flavonoids, and steroids extracted from medicinal plants [2, 21, 31, 53–58]. We can therefore infer that the analgesic effects displayed by the extracts may be caused by the presence of these previously described phytoconstituents and now these phytoconstituents were also recognized in our samples.

The thiopental-induced sedation test revealed that the tested extracts considerably lowered the sleep latency and increased sleep duration in the test animals in a dose-responsive way (Table 8). An indication of the extracts under investigation's sedative potential was the extension of thiopental-induced sleep. Dopamine, serotonin, opioid, GABA, and GABA-BDZ receptor complexes are known to play a role in the extension of sleep [59]. As a result, it can be assumed that the extracts under investigation might extend thiopental-induced sedation by interacting with one or more of the aforementioned receptors. Furthermore, a number of neurotransmitter systems, including acetylcholine (ACh), dopamine, serotonin, GABA, opioid, and noradrenaline, modulate spontaneous locomotor activity and rotarod performance (skeletal muscle relaxation) [60–62]. The current investigation demonstrated that, in a dose-modulated way, all extracts significantly (*p* < 0.05;  *p* < 0.01) decreased rotarod performance (Table 9). Therefore, it may be concluded that examined extracts may have acted on the dual receptor complex of GABA and opioid receptors to create inhibitory effects on locomotor activity and rotarod performance. According to studies, plant extracts high in terpenoids, saponins, alkaloids, and glycosides have sedative and anxiolytic characteristics that interfere with their actions at the benzodiazepine site of the GABAergic complex structure or act as immediate or aberrant modulators that are in charge of increases in GABA activity in the brain that result in drowsiness and facies [60–65]. More research is required to pinpoint the particular phytoconstituents responsible for the neuropharmacology activities and the related mechanisms of action.

In clinical and research settings, the oral glucose tolerance test is a crucial tool for determining insulin release and insulin resistance. The “standard of excellence” for identifying diabetes mellitus is a test known as the oral glucose tolerance test, or OGTT. In cases of reduced glucose tolerance, the body takes a lot of time to eliminate the difficult glucose. The maintenance of blood glucose homeostasis is hampered by the reduction of glucose tolerance because it reduces the absorption of glucose by muscle and fat [33, 66, 67]. This method is referred to as the physiological induction of diabetes mellitus since it temporarily raises the animal's blood glucose level without harming the pancreas [66]. So, the effect of the investigated extracts on glucose homeostasis was assessed using the oral glucose tolerance test. Compared to the reference drug metformin, the crude methanolic extracts of seeds and fruits and their fractions considerably reduced blood sugar levels in mice's OGTT test over the course of up to 2 h (*p* < 0.05; *p* < 0.01; Table 10). According to previous investigations, plant extracts reduced blood glucose levels by boosting insulin secretion, improving insulin sensitivity and glucose uptake at peripheral tissue, increasing glucose excretion, and blocking glycogenolysis and gluconeogenesis [13, 68]. The precise mechanism of action is unknown, but the investigated extracts' ability to lower blood sugar may be due to increased insulin secretion, improved insulin sensitivity and glucose uptake in peripheral tissues, increased glucose excretion, and inhibition of glycogenolysis and gluconeogenesis [66, 67]. The effect of the test extracts, however, was very significant (*p* < 0.01) at the higher dose of 600 mg/kg, indicating that the extracts under investigation had reached the maximum level of their antihyperglycemic action. This may be because the test extracts have a sufficient amount of active phytochemicals at this dosage.

The effectiveness of the investigated plant extracts as antidiarrheal agents was assessed using castor oil and magnesium sulfate-induced diarrheal models. Dietary macronutrients and phytochemical content, some particular foods (fruits and oilseeds), and the diet as a whole have the capacity to quickly alter the gut microbiota [68]. Castor oil has a long history of being used to cause diarrhea [69]. There are a number of theories put forth to explain why castor oil causes diarrhea: (i) intestinal lipases produce its active metabolite ricinoleic acid which has irritating laxative action by creating localized irritation and inflammation of the intestinal mucosa, which leads to the release of prostaglandins and ultimately increases gastrointestinal motility, net secretion of water, and electrolytes; (ii) it also inhibits intestinal Na+/K + -ATPase activity, which reduces normal fluid absorption; and (iii) it activates adenylate cyclase or mucosal cAMP-mediated active secretion and nitric oxide [70, 71]. These provoked us to utilize castor oil to cause diarrhea in this study because it is comparable to the pathophysiology of diarrhea. The studied extracts demonstrated an antidiarrheal effect at the test dosages used, as shown by a significantly (*p* < 0.05; *p* < 0.01) delayed onset of diarrhea and decreased number of fecal matter frequency (number of wet feces) (Table 11). The examined extracts' ability to increase fluid and electrolyte absorption through the gastrointestinal system is one of the possible explanations for their antidiarrheal effectiveness. Again, previous reports showed that magnesium sulfate causes diarrhea because of its osmotic characteristics, which prevent water ions from being reabsorbed and increase the volume of the intestinal contents. In addition, this salt encourages the release of cholecystokinin from the duodenal mucosa, which boosts secretions. Furthermore, it inhibits the reabsorption of water and sodium chloride as well as has a motor action on the small intestine [70]. The studied extracts (PFJM, PFJE, PFJC, SFJM, SFJE, and SFJC) significantly reduced diarrhea brought on by magnesium sulfate (Table 12). Since the extracts slowed the gastrointestinal transit in mice compared to the negative control, they may increase the absorption of water and electrolytes from the gastrointestinal tract. Their antidiarrheal efficacy may have been enhanced, at least in part, by the extract's induction of a delay in the gastrointestinal transit, which gave more time for absorption. The percentage of inhibition was significantly (*p* < 0.05; *p* < 0.01) increased at all trial doses in comparison to the standard, and the quantity of diarrheal feces was also noticeably reduced. The tested extracts significantly (*p* < 0.01; *p* < 0.05) increased the reabsorption of water in the gastrointestinal transit test and decreased the intestinal transit of charcoal meal, indicating a dose-dependent antimotility effect. This allows for the absorption of water and electrolytes, which in turn has an antidiarrheal effect (Table 13). All of these findings suggest that the antisecretory properties of extracts may be related to the presence of flavonoids, tannins, terpenoids, and saponins as well as to the synergistic interactions between these compounds [70–74].

*F. jangomas* fruit has been used for centuries to treat a wide range of human ailments in Bangladesh [7–12]. Our recent study also demonstrated that the aforementioned plant's fruit and seed are rich in secondary bioactive components with a range of health-promoting effects, such as antiarthritic, thrombolytic, anthelmintic, analgesic, CNS depressant, antidiarrheal, and hypoglycemic features. Further investigation is necessary to elucidate the precise mechanism and bioactive components.

## 5. Conclusion

The traditional uses of the fruit and seed of *F. jangomas* as antiarthritic, thrombolytic, anthelmintic, analgesic, CNS depressing, antidiarrheal, and hypoglycemic medicines are validated by our current findings. The research backs up the conventional theories as well; nevertheless, more investigation is required to pinpoint the precise chemical components that give rise to the previously listed characteristics. We will carry out additional studies to identify the bioactive compounds and comprehend the exact molecular mechanisms in order to develop a safe and effective dosage and to confirm the likelihood of its usage in the prevention and treatment of various diseases.

## Figures and Tables

**Table 1:** Treatment design.

Group name	Treatment
Group I negative control	Vehicle (1% tween 80 + normal saline) ^*∗*^(2% w/v of gum acacia with normal saline) only for the tail immersion test
Group II positive control	Standard (1 mg/kg of diazepam for motor coordination and thiopental-induced sleeping experiments, 25 mg/kg of diclofenac sodium for the writhing test, 2 mg/kg morphine for the tail immersion test, 10 mg/kg of metformin for the hypoglycemic test, and 2 mg/kg of loperamide for castor oil- and magnesium sulfate-induced diarrheal assessments) drug for the respective test
Group III	PFJM (200 mg/kg)
Group IV	PFJM (400 mg/kg)
Group V	PFJM (600 mg/kg)
Group VI	PFJE (200 mg/kg)
Group VII	PFJE (400 mg/kg)
Group VIII	PFJE (600 mg/kg)
Group IX	PFJC (200 mg/kg)
Group X	PFJC (400 mg/kg)
Group XI	PFJC (600 mg/kg)
Group XII	SFJM (200 mg/kg)
Group XIII	SFJM (400 mg/kg)
Group XIV	SFJM (600 mg/kg)
Group XV	SFJE (200 mg/kg)
Group XVI	SFJE (400 mg/kg)
Group XVII	SFJE (600 mg/kg)
Group XVIII	SFJC (200 mg/kg)
Group XIX	SFJC (400 mg/kg)
Group XX	SFJC (600 mg/kg)

^*∗*^For only tail immersion test, the negative control group was treated with 2% w/v of Gum acacia in normal saline.

**Table 2:** Table of quantitative phytochemicals of *F. jangomas* extracts.

Samples	Flavonoid (%)	Phenol (mg/gm)	Alkaloid (%)	Tannin (mg/gm)	Saponin (%)	Cyanogenic glycoside (mg/100g)	Lipid (%)	Carbohydrate (%)	Protein (%)
PFJM	12.26 ± 0.37	8.20 ± 0.37	7.63 ± 0.25	8.50 ± 0.30	2.03 ± 0.15	550.00 ± 1.53	0.30 ± 0.15	20.27 ± 0.37	2.03 ± 0.66
PFJE	13.51 ± 0.28	3.17 ± 0.16	1.80 ± 0.20	5.33 ± 0.16	3.50 ± 0.20	481.00 ± 2.00	0.20 ± 0.66	22.51 ± 0.29	1.03 ± 0.15
PFJC	14.80 ± 0.20	8.50 ± 0.10	11.66 ± 0.15	12.63 ± 0.21	11.86 ± 0.06	528.00 ± 2.52	0.50 ± 0.26	19.63 ± 0.33	4.53 ± 0.32
SFJM	14.83 ± 0.21	8.93 ± 0.15	10.80 ± 0.25	12.90 ± 0.10	11.00 ± 0.26	537.67 ± 2.08	0.10 ± 0.33	18.96 ± 0.26	5.63 ± 0.66
SFJE	12.26 ± 0.37	6.40 ± 0.40	7.37 ± 0.57	12.60 ± 0.06	7.67 ± 0.26	429.33 ± 1.52	4.50 ± 0.20	8.63 ± 0.15	2.67 ± 0.74
SFJC	12.40 ± 0.32	8.96 ± 0.21	12.07 ± 0.15	13.30 ± 0.17	11.00 ± 0.74	678.23 ± 0.68	5.00 ± 0.74	23.51 ± 0.217	17.96 ± 0.17

Values are the mean of three independent analyses ±SD (*n* = 3).

**Table 3:** *In vitro* antiarthritic efficacy of *F. jangomas* extracts.

Sample	% of inhibition				
	Concentration (*μ*g/mL)				
	100	200	300	400	500
Standard (diclofenac sodium)	80.61 ± 0.94	83.33 ± 0.48	83.73 ± 0.89	85.06 ± 1.80	88.46 ± 0.73
PFJM	57.98 ± 2.20^a^^*∗*^	60.02 ± 3.74^b^^*∗∗*^	60.87 ± 3.95^a^^*∗*^	64.26 ± 1.84^b^^*∗∗*^	65.91 ± 0.50^a^^*∗*^
PFJE	63.33 ± 2.64^a^^*∗*^	65.55 ± 0.61^a^^*∗*^	66.35 ± 1.92^a^^*∗*^	66.68 ± 0.42^a^^*∗*^	67.89 ± 0.18^a^^*∗*^
PFJC	68.29 ± 1.06^b^^*∗∗*^	69.63 ± 0.91^b^^*∗∗*^	72.15 ± 1.12^b^^*∗∗*^	72.95 ± 0.76^b^^*∗∗*^	74.28 ± 1.16^b^^*∗∗*^
SFJM	59.71 ± 1.47^a^^*∗*^	60.42 ± 1.90^b^^*∗∗*^	61.66 ± 2.06^b^^*∗∗*^	62.50 ± 2.04^b^^*∗∗*^	63.80 ± 1.42^a^^*∗*^
SFJE	66.11 ± 0.93^b^^*∗∗*^	67.11 ± 0.93^b^^*∗∗*^	67.87 ± 0.57^a^^*∗*^	69.19 ± 0.98^a^^*∗*^	71.72 ± 0.41^b^^*∗∗*^
SFJC	70.95 ± 0.58^a^^*∗*^	71.95 ± 1.49^b^^*∗∗*^	73.88 ± 0.67^b^^*∗∗*^	75.59 ± 0.46^b^^*∗∗*^	79.25 ± 0.83^b^^*∗∗*^

PFJM, methanolic extract of the fruit; PFJE, ethyl acetate extract of the fruit; PFJC, chloroform extract of the fruit; SFJM, methanolic extract of the seed; SFJE, ethyl acetate extract of the seed; SFJC, chloroform extract of the seed. Results are reported as the mean standard deviation of triplicate measurements (mean ± SEM; *n* = 3) and analyzed by one-way analysis of variance (ANOVA) trailed by Dunnett's test; ^a^^*∗*^*p* < 0.05 and ^b^^*∗∗*^*p* < 0.01 are significant compared to the standard drug (diclofenac sodium).

**Table 4:** *In vitro* anti-inflammatory efficacy of *F. jangomas* extracts.

Sample	% hemolysis					% protection				
	Concentration (*μ*g/mL)					Concentration (*μ*g/mL)				
	1000	2000	3000	4000	5000	1000	2000	3000	4000	5000
Standard (indomethacin)	87.25 ± 0.85	71.37 ± 0.1.39	52.15 ± 1.15	33.92 ± 1.03	17.65 ± 0.58	15.60 ± 1.59	27.27 ± 0.69	42.27 ± 0.91	65.91 ± 1.36	83.03 ± 0.95
PFJM	50.98 ± 1.22^a^^*∗*^	45.59 ± 1.28^b^^*∗∗*^	43.82 ± 0.29^a^^*∗*^	40.09 ± 0.44^b^^*∗∗*^	34.70 ± 0.59^a^^*∗*^	45.60 ± 1.39^a^^*∗*^	51.51 ± 0.69^a^^*∗*^	55.30 ± 0.94	60.46 ± 1.20^b^^*∗∗*^	69.99 ± 0.78^a^^*∗*^
PFJE	53.63 ± 1.03^a^^*∗*^	43.04 ± 0.89^a^^*∗*^	38.92 ± 0.44^a^^*∗*^	32.94 ± 0.59^a^^*∗*^	27.84 ± 1.03^a^^*∗*^	46.21 ± 0.26^a^^*∗*^	49.96 ± 1.36^a^^*∗*^	56.36 ± 1.82^a^^*∗*^	63.03 ± 1.05^a^^*∗*^	71.66 ± 1.15^a^^*∗*^
PFJC	56.66 ± 1.33^b^^*∗∗*^	46.47 ± 0.78^b^^*∗∗*^	34.21 ± 1.38^b^^*∗∗*^	25.94 ± 0.49^b^^*∗∗*^	22.06 ± 1.06^a^^*∗*^	52.12 ± 0.69^b^^*∗∗*^	63.48 ± 0.69^b^^*∗∗*^	66.67 ± 0.95^a^^*∗*^	68.93 ± 0.52^a^^*∗*^	73.18 ± 0.04^a^^*∗*^
SFJM	51.27 ± 1.19	44.90 ± 0.89^a^^*∗*^	40.68 ± 1.19^a^^*∗*^	37.74 ± 0.44^a^^*∗*^	31.67 ± 1.18^a^^*∗*^	47.88 ± 1.14	51.97 ± 0.94^a^^*∗*^	58.79 ± 0.52^a^^*∗*^	64.69 ± 0.52^a^^*∗*^	74.39 ± 0.94^a^^*∗*^
SFJE	57.19 ± 1.07^a^^*∗*^	40.98 ± 1.40^a^^*∗*^	35.49 ± 1.60^a^^*∗*^	28.23 ± 1.06^a^^*∗*^	25.69 ± 1.39^a^^*∗*^	47.73 ± 1.21^a^^*∗*^	50.42 ± 0.81^a^^*∗*^	60.61 ± 1.05^a^^*∗*^	65.61 ± 1.89^a^^*∗*^	76.36 ± 0.79^b^^*∗∗*^
SFJC	66.73 ± 1.39^a^^*∗*^	60.29 ± 1.47^a^^*∗*^	50.58 ± 0.88^a^^*∗*^	39.71 ± 0.88^a^^*∗*^	19.22 ± 1.32^b^^*∗∗*^	58.40 ± 0.96^a^^*∗*^	64.39 ± 0.52^b^^*∗∗*^	68.93 ± 0.53^a^^*∗*^	73.48 ± 0.25^a^^*∗*^	77.43 ± 0.52a^*∗*^

PFJM, methanolic extract of the fruit; PFJE, ethyl acetate extract of the fruit; PFJC, chloroform extract of the fruit; SFJM, methanolic extract of the seed; SFJE, ethyl acetate extract of the seed; SFJC, chloroform extract of the seed. Results are reported as the mean standard deviation of triplicate measurements (mean ± SEM; *n* = 3) and analyzed by one-way analysis of variance (ANOVA) trailed by Dunnett's test; ^a^^*∗*^*p* < 0.05 and ^b^^*∗∗*^*p* < 0.01 are significant compared to the standard drug (indomethacin).

**Table 5:** Anthelmintic activity of *F. jangomas extracts* at diverse concentrations.

Treatment	Conc. used (mg/mL)	Time taken for paralysis (min) *X* ± SD	Time taken for death (min) *X* ± SD
Negative control		—	—

Positive control albendazole	20	28.67 ± 1.15	40.33 ± 1.52

PFJM	25	66.67 ± 1.15^a^^*∗*^	76.33 ± 2.08^a^^*∗*^
	50	61.67 ± 1.15^a^^*∗*^	72.67 ± 2.527^a^^*∗*^
	75	59.67 ± 0.58^b^^*∗∗*^	66.33 ± 1.53^b^^*∗∗*^

PFJE	25	61.33 ± 1.53^a^^*∗*^	70.33 ± 2.53^b^^*∗∗*^
	50	57.33 ± 1.53^b^^*∗∗*^	64.33 ± 2.52^b^^*∗∗*^
	75	44.33 ± 2.53^a^^*∗*^	48.33 ± 2.08^a^^*∗*^

PFJC	25	60.67 ± 1.53^a^^*∗*^	67.33 ± 1.52^a^^*∗*^
	50	56 ± 1.73^a^^*∗*^	59.67 ± 1.52^a^^*∗*^
	75	37.67 ± 1.15^b^^*∗∗*^	45.67 ± 1.52^b^^*∗∗*^

SFJM	25	62.67 ± 1.52	72.33 ± 2.51
	50	58.33 ± 0.577^a^^*∗*^	67.33 ± 2.08^a^^*∗*^
	75	50.67 ± 2.51^b^^*∗∗*^	64.33 ± 1.52^b^^*∗*^

SFJE	25	57.67 ± 1.15^a^^*∗*^	62.67 ± 2.51^b^^*∗∗*^
	50	56.67 ± 1.15^b^^*∗∗*^	63.33 ± 2.52^b^^*∗∗*^
	75	39.33 ± 0.577^b^^*∗∗*^	44.67 ± 3.51^b^^*∗∗*^

SFJC	25	55.67 ± 0.58^b^^*∗∗*^	59.33 ± 1.53^b^^*∗∗*^
	50	53.33 ± 1.52^a^^*∗*^	57.33 ± 2.08^b^^*∗∗*^
	75	33.33 ± 1.52^b^^*∗∗*^	40.67 ± 2.08^b^^*∗∗*^

PFJM, methanolic extract of the fruit; PFJE, ethyl acetate extract of the fruit; PFJC, chloroform extract of the fruit; SFJM, methanolic extract of the seed; SFJE, ethyl acetate extract of the seed; SFJC, chloroform extract of the seed. Results are reported as the mean standard deviation of triplicate measurements (mean ± SEM; *n* = 3) and analyzed by one-way analysis of variance (ANOVA) trailed by Dunnett's test; ^a^^*∗*^*p* < 0.05 and ^b^^*∗∗*^*p* < 0.01 are significant compared to the positive control (albendazole).

**Table 6:** Analgesic effect of *F. jangomas* extracts in the acetic acid-induced writhing model.

Group	Treatment	Number of writhing	% of inhibition
Negative control (I)	Tween 80 solution	25.83 ± 2.64	—
Standard (II)	Diclofenac sodium 25 mg/kg	6.83 ± 1.47^a^^*∗∗*^	73.56
III	PFJM (200 mg/kg)	17.83 ± 3.06^a^^*∗*^	30.97
IV	PFJM (400 mg/kg)	13.83 ± 2.64^a^^*∗*^^b^^*∗*^	46.46
V	PFJM (600 mg/kg)	11.67 ± 1.21^a^^*∗∗*^^b^^*∗*^	54.82
VI	PFJE (200 mg/kg)	15.83 ± 1.47^a^^*∗*^^b^^*∗*^	38.71
VII	PFJE (400 mg/kg)	13.17 ± 1.72^a^^*∗*^	49.01
VIII	PFJE (600 mg/kg)	10.83 ± 1.84^a^^*∗∗*^^b^^*∗*^	58.07
IX	PFJC (200 mg/kg)	15.17 ± 1.17^a^^*∗*^	41.27
X	PFJC (400 mg/kg)	12.33 ± 1.21^a^^*∗*^^b^^*∗*^	52.26
XI	PFJC (600 mg/kg)	9.67 ± 0.82^a^^*∗∗*^^b^	62.56
XII	SFJM (200 mg/kg)	16.67 ± 1.03^a^^*∗*^^b^^*∗*^	35.46
XIII	SFJM (400 mg/kg)	13.00 ± 2.97^a^^*∗*^	49.67
XIV	SFJM (600 mg/kg)	10.83 ± 1.84^a^^*∗*^^b^^*∗∗*^	58.07
XV	SFJE (200 mg/kg)	14.5 ± 0.55^a^^*∗*^^b^	43.86
XVI	SFJE (400 mg/kg)	11.83 ± 1.47^a^^*∗*^^b^^*∗∗*^	54.20
XVII	SFJE (600 mg/kg)	9.83 ± 1.94^a^^*∗*^^b^^*∗∗*^	61.94
XVIII	SFJC (200 mg/kg)	13.17 ± 1.47^a^^*∗*^^b^^*∗*^	49.01
XIX	SFJC (400 mg/kg)	11.67 ± 1.21^a^^*∗∗*^^b^^*∗∗*^	54.82
XX	SFJC (600 mg/kg)	8.67 ± 0.82^a^^*∗∗*^^b^^*∗∗*^	66.43

PFJM, methanolic extract of the fruit; PFJE, ethyl acetate extract of the fruit; PFJC, chloroform extract of the fruit; SFJM, methanolic extract of the seed; SFJE, ethyl acetate extract of the seed; SFJC, chloroform extract of the seed. When compared to the control group, the data values are shown as mean SEM (*n* = 6); ^a^^*∗*^*p* < 0.05 and ^a^^*∗∗*^*p* < 0.01 compared to the control group; ^b^^*∗*^*p* < 0.05 and ^b^^*∗∗*^*p* < 0.01 compared to the standard group (one-way ANOVA with post hoc Dunnett's test).

**Table 7:** Analgesic effect of *F. jangomas* extracts in the tail immersion test.

Group	Treatment	Response time (sec ± SEM)			
		Pretreatment	30 min	60 min	90 min
Negative control (I)	2% w/v of gum acacia with normal saline	1.64 ± 0.03	1.70 ± 0.04	1.71 ± 0.01	1.74 ± 0.02
Standard (II)	Morphine 2 mg/kg	2.11 ± 0.07^b^^*∗∗*^	8.98 ± 0.09^b^^*∗∗*^	5.99 ± 0.08^b^^*∗∗*^	3.94 ± 0.08^b^^*∗∗*^
III	PFJM (200 mg/kg)	1.73 ± 0.03	1.80 ± 0.05^a^^*∗*^	3.70 ± 0.04^a^^*∗*^^b^^*∗*^	2.36 ± 0.04^a^^*∗*^
IV	PFJM (400 mg/kg)	1.76 ± 0.05	2.23 ± 0.07	4.17 ± 0.12^a^^*∗∗*^^b^^*∗*^	3.48 ± 0.22^a^^*∗∗*^
V	PFJM (600 mg/kg)	1.97 ± 0.03^a^^*∗∗*^	3.45 ± 0.18^a^^*∗∗*^	5.60 ± 0.22^a^^*∗∗*^^b^^*∗*^	4.46 ± 0.24^a^^*∗∗*^^b^^*∗∗*^
VI	PFJE (200 mg/kg)	1.85 ± 0.03^a^^*∗∗*^	2.18 ± 0.06	3.80 ± 0.05^a^^*∗∗*^	2.47 ± 0.07^a^^*∗∗*^
VII	PFJE (400 mg/kg)	1.84 ± 0.03^a^^*∗∗*^	2.30 ± 0.05	4.24 ± 0.09^a^^*∗∗*^^b^^*∗*^	3.57 ± 0.12^a^^*∗∗*^
VIII	PFJE (600 mg/kg)	1.97 ± 0.03^b^^*∗∗*^	3.45 ± 0.18^a^^*∗*^	5.68 ± 0.^11a^^*∗∗*^^b^^*∗*^	4.46 ± 0.24^a^^*∗∗*^^b^^*∗*^
IX	PFJC (200 mg/kg)	1.98 ± 0.08^a^^*∗*^	2.31 ± 0.08	4.03 ± 0.05^a^^*∗∗*^	2.66 ± 0.06
X	PFJC (400 mg/kg)	2.08 ± 0.03^a^^*∗*^^b^^*∗*^	2.82 ± 0.06^a^^*∗∗*^	4.24 ± 0.09^a^^*∗∗*^	3.75 ± 0.12^a^^*∗∗*^
XI	PFJC (600 mg/kg)	1.95 ± 0.06^a^^*∗∗*^^b^^*∗*^	3.59 ± 0.09^a^^*∗∗*^^b^^*∗*^	6.02 ± 0.17^a^^*∗*^	4.67 ± 0.19^a^^*∗∗*^^b^^*∗*^
XII	SFJM (200 mg/kg)	1.85 ± 0.05^a^^*∗*^	2.01 ± 0.12	3.81 ± 0.12^a^^*∗*^	2.32 ± 0.08^a^^*∗∗*^
XIII	SFJM (400 mg/kg)	1.90 ± 0.08^a^^*∗∗*^	2.29 ± 0.11	4.41 ± 0.11^a^^*∗*^^b^^*∗*^	3.62 ± 0.17^a^^*∗∗*^^b^^*∗*^
XIV	SFJM (600 mg/kg)	2.04 ± 0.10^a^^*∗∗*^^b^^*∗*^	3.83 ± 0.17^a^^*∗∗*^^b^^*∗∗*^	5.80 ± 0.24^a^^*∗∗*^^b^^*∗*^	4.69 ± 0.27^a^^*∗∗*^
XV	SFJE (200 mg/kg)	2.02 ± 0.09^a^^*∗∗*^	2.26 ± 0.07	3.59 ± 0.17	2.66 ± 0.17
XVI	SFJE (400 mg/kg)	2.04 ± 0.14^a^^*∗*^	2.49 ± 0.12^a^^*∗*^	4.49 ± 0.31^a^^*∗∗*^	3.66 ± 0.24^a^^*∗∗*^
XVII	SFJE (600 mg/kg)	2.05 ± 0.17^a^^*∗∗*^^b^^*∗*^	3.62 ± 0.29^a^^*∗∗*^^b^^*∗*^	6.05 ± 0.12^a^^*∗∗*^	4.60 ± 0.26^a^^*∗∗*^
XVIII	SFJC (200 mg/kg)	2.12 ± 0.12^a^^*∗∗*^	2.41 ± 0.13	4.33 ± 0.39^a^^*∗∗*^	2.78 ± 0.27
XIX	SFJC (400 mg/kg)	2.16 ± 0.11^a^^*∗∗*^^b^^*∗*^	3.17 ± 0.43^a^^*∗∗*^	4.47 ± 0.38^a^^*∗∗*^^b^^*∗∗*^	3.95 ± 0.28^a^^*∗∗*^
XX	SFJC (600 mg/kg)	2.18 ± 0.11^a^^*∗∗*^	4.04 ± 0.20^a^^*∗∗*^^b^^*∗∗*^	6.49 ± 0.51^a^^*∗∗*^^b^^*∗*^	5.00 ± 0.29^a^^*∗∗*^

PFJM, methanolic extract of the fruit; PFJE, ethyl acetate extract of the fruit; PFJC, chloroform extract of the fruit; SFJM, methanolic extract of the seed; SFJE, ethyl acetate extract of the seed; SFJC, chloroform extract of the seed. When compared to the control group, the data values are shown as mean SEM (*n* = 5); ^a^^*∗*^*p* < 0.05 and ^a^^*∗∗*^*p* < 0.01 compared to the control group; ^b^^*∗*^p < 0.05 and ^b^^*∗∗*^*p* < 0.01 compared to the standard group (one-way ANOVA with post hoc Dunnett's test).

**Table 8:** Effect of *F. jangomas* extracts on thiopental sodium-induced hypnosis.

Group	Treatment	Onset of sleep (min)	Duration of sleep (min)
Negative control (I)	Tween 80 solution	5.66 ± 0.59	25.4 ± 2.30
Standard (II)	Diazepam 1 mg/kg	3.86 ± 0.56^a^^*∗∗*^	95.6 ± 3.91^a^^*∗∗*^
III	PFJM (200 mg/kg)	5.32 ± 0.30^a^^*∗*^	31.6 ± 2.30^a^^*∗∗*^
IV	PFJM (400 mg/kg)	4.90 ± 0.52^a^^*∗*^	44.4 ± 3.78^*∗*^
V	PFJM (600 mg/kg)	4.72 ± 0.41^a^^*∗*^^b^^*∗*^	71.6 ± 2.70^*∗*^
VI	PFJE (200 mg/kg)	5.30 ± 0.23^a^^*∗*^	32.4 ± 1.82^a^^*∗∗*^
VII	PFJE (400 mg/kg)	4.84 ± 0.59	45.2 ± 3.77^*∗*^
VIII	PFJE (600 mg/kg)	5.02 ± 0.31^a^^*∗*^^b^^*∗*^	72.6 ± 1.52^a^^*∗∗*^^b^^*∗∗*^
IX	PFJC (200 mg/kg)	5.02 ± 0.53^a^^*∗*^	34.4 ± 1.82^a^^*∗∗*^^b^^*∗*^
X	PFJC (400 mg/kg)	4.66 ± 0.61^a^^*∗∗*^	49.8 ± 3.11^a^^*∗∗*^
XI	PFJC (600 mg/kg)	4.56 ± 0.43^a^^*∗*^	75.4 ± 2.30^a^^*∗∗*^^b^^*∗*^
XII	SFJM (200 mg/kg)	5.54 ± 0.95^a^^*∗*^^b^^*∗*^	38.6 ± 2.07^a^^*∗∗*^
XIII	SFJM (400 mg/kg)	5.02 ± 1.15^a^^*∗*^	59.2 ± 2.77^a^^*∗∗*^
XIV	SFJM (600 mg/kg)	4.48 ± 0.31^a^^*∗∗*^	75.6 ± 3.36^a^^*∗∗*^^b^^*∗∗*^
XV	SFJE (200 mg/kg)	5.30 ± 0.79^a^^*∗*^	39.2 ± 1.92^a^^*∗∗*^
XVI	SFJE (400 mg/kg)	4.90 ± 1.05^a^^*∗*^	61.6 ± 1.67^a^^*∗∗*^
XVII	SFJE (600 mg/kg)	4.28 ± 0.19^a^^*∗∗*^^b^^*∗∗*^	80.2 ± 1.64^a^^*∗∗*^^b^^*∗*^
XVIII	SFJC (200 mg/kg)	5.04 ± 0.55^a^^*∗*^	43.4 ± 2.30^a^^*∗∗*^
XIX	SFJC (400 mg/kg)	4.62 ± 0.63^a^^*∗*^^b^^*∗*^	63.8 ± 2.77^a^^*∗∗*^
XX	SFJC (600 mg/kg)	4.18 ± 0.24^a^^*∗∗*^	85.2 ± 2.39^a^^*∗∗*^^b^^*∗∗*^

PFJM, methanolic extract of the fruit; PFJE, ethyl acetate extract of the fruit; PFJC, chloroform extract of the fruit; SFJM, methanolic extract of the seed; SFJE, ethyl acetate extract of the seed; SFJC, chloroform extract of the seed. When compared to the control group, the data values are shown as mean SEM (*n* = 5); ^a^^*∗*^*p* < 0.05 and ^a^^*∗∗*^*p* < 0.01 compared to the control group; ^b^^*∗*^*p* < 0.05 and ^b^^*∗∗*^*p* < 0.01 compared to the standard group (one-way ANOVA with post hoc Dunnett's test).

**Table 9:** Effect of *F. jangomas* extracts on motor coordination in the rotarod test.

Group	Treatment	Response	
		Rotarod performance (sec)	% of inhibition
Negative control (I)	Tween 80 solution	122.4 ± 3.05	—
Standard (II)	Diazepam 1 mg/kg	28.2 ± 1.304^a^^*∗∗*^	76.96
III	PFJM (200 mg/kg)	115.6 ± 3.507^a^^*∗*^	5.55
IV	PFJM (400 mg/kg)	83 ± 3.162^a^^*∗*^	32.18
V	PFJM (600 mg/kg)	63.2 ± 4.207^a^^*∗∗*^^b^^*∗*^	48.37
VI	PFJE (200 mg/kg)	113.8 ± 2.280^a^^*∗*^	7.03
VII	PFJE (400 mg/kg)	73.2 ± 3.271^a^^*∗∗*^^b^^*∗*^	40.19
VIII	PFJE (600 mg/kg)	52.8 ± 3.194^a^^*∗∗*^^b^^*∗∗*^	56.86
IX	PFJC (200 mg/kg)	107.4 ± 4.827^a^^*∗*^	12.25
X	PFJC (400 mg/kg)	69.2 ± 1.924^a^^*∗*^^b^^*∗*^	43.46
XI	PFJC (600 mg/kg)	47.6 ± 2.302^a^^*∗∗*^^b^^*∗∗*^	61.11
XII	SFJM (200 mg/kg)	113.8 ± 3.114^a^^*∗*^	7.026
XIII	SFJM (400 mg/kg)	79.4 ± 1.140^a^^*∗*^	35.13
XIV	SFJM (600 mg/kg)	61.2 ± 1.924^a^^*∗∗*^^b^^*∗∗*^	50.00
XV	SFJE (200 mg/kg)	108.6 ± 1.342^a^^*∗*^	11.27
XVI	SFJE (400 mg/kg)	68.2 ± 4.087^a^^*∗*^	44.28
XVII	SFJE (600 mg/kg)	51.8 ± 2.588^a^^*∗∗*^^b^^*∗*^	57.68
XVIII	SFJC (200 mg/kg)	100.6 ± 3.647^a^^*∗∗*^	17.81
XIX	SFJC (400 mg/kg)	59.8 ± 2.683^a^^*∗∗*^^b^^*∗*^	51.14
XX	SFJC (600 mg/kg)	45.4 ± 2.408^a^^*∗∗*^^b^^*∗∗*^	62.91

PFJM, methanolic extract of the fruit; PFJE, ethyl acetate extract of the fruit; PFJC, chloroform extract of the fruit; SFJM, methanolic extract of the seed; SFJE, ethyl acetate extract of the seed; SFJC, chloroform extract of the seed. When compared to the control group, the data values are shown as mean SEM (*n* = 5); ^a^^*∗*^*p* < 0.05 and ^a^^*∗∗*^*p* < 0.01 compared to the control group; ^b^^*∗*^*p* < 0.05 and ^b^^*∗∗*^*p* < 0.01 compared to the standard group (one-way ANOVA with post hoc Dunnett's test).

**Table 10:** Blood glucose level in oral glucose tolerance test of *F. jangomas* extracts.

Group	Treatment	Blood Glucose level			
		0 min	30 min	1 h	2 h
Negative control (I)	Tween 80 solution	7.3 ± 0.47	23.64 ± 1.58	20.96 ± 0.94	17.14 ± 0.96
Standard (II)	Metformin 10 mg/kg	6.88 ± 0.83	15.84 ± 0.57^a^^*∗∗*^^b^^*∗*^	6.56 ± 0.37^a^^*∗∗*^^b^^*∗∗*^	5.42 ± 0.40^a^^*∗∗*^
III	PFJM (200 mg/kg)	6.84 ± 0.31	19.64 ± 0.60^a^^*∗*^	16.08 ± 0.58^a^^*∗∗*^	11.4 ± 0.53^a^^*∗∗*^
IV	PFJM (400 mg/kg)	6.64 ± 0.34^a^^*∗*^	17.56 ± 0.78^a^^*∗*^	13.88 ± 0.79^a^^*∗∗*^^b^^*∗*^	10.36 ± 0.42^a^^*∗∗*^
V	PFJM (600 mg/kg)	6.1 ± 0.31^a^^*∗∗*^	14.16 ± 0.42^*∗∗*^	9.98 ± 0.22^a^^*∗∗*^	8.58 ± 0.41^a^^*∗∗*^
VI	PFJE (200 mg/kg)	6.78 ± 0.24	18.62 ± 0.66^a^^*∗*^	15.38 ± 0.37^a^^*∗∗*^	10.98 ± 0.71^a^^*∗∗*^^b^^*∗∗*^
VII	PFJE (400 mg/kg)	6.5 ± 0.19^a^^*∗*^	17.08 ± 1.01^a^^*∗*^	13.8 ± 0.27^a^^*∗∗*^	9.96 ± 0.19^a^^*∗∗*^^b^^*∗*^
VIII	PFJE (600 mg/kg)	5.9 ± 0.12^a^^*∗∗*^	13.76 ± 0.29^a^^*∗∗*^	9.88 ± 0.15^a^^*∗∗*^	8.12 ± 0.25^a^^*∗∗*^
IX	PFJC (200 mg/kg)	6.76 ± 0.32	18.62 ± 0.66^a^^*∗*^	15.38 ± 0.37^a^^*∗*^	10.98 ± 0.71^a^^*∗∗*^
X	PFJC (400 mg/kg)	5.94 ± 0.32^a^^*∗*^	16.28 ± 0.86^a^^*∗*^	13.52 ± 0.26^a^^*∗∗*^^b^^*∗*^	9.96 ± 0.19^a^^*∗∗*^
XI	PFJC (600 mg/kg)	5.66 ± 0.27^a^^*∗∗*^	12.92 ± 0.58^a^^*∗∗*^	8.96 ± 0.55^a^^*∗∗*^	7.68 ± 0.49^a^^*∗∗*^
XII	SFJM (200 mg/kg)	6.68 ± 0.56	19.22 ± 0.46^a^^*∗∗*^	15.82 ± 0.51^a^^*∗∗*^	11.22 ± 0.36^a^^*∗∗*^^b^^*∗*^
XIII	SFJM (400 mg/kg)	6.5 ± 0.35^a^^*∗∗*^	16.8 ± 0.21^a^^*∗∗*^	13.2 ± 0.35^a^^*∗∗*^	9.94 ± 0.11^a^^*∗∗*^
XIV	SFJM (600 mg/kg)	5.86 ± 0.18^a^^*∗∗*^	13.94 ± 0.47^a^^*∗∗*^	9.78 ± 0.08^a^^*∗∗*^	8.28 ± 0.13^a^^*∗∗*^
XV	SFJE (200 mg/kg)	6.62 ± 0.21^a^^*∗*^	18.4 ± 0.22^a^^*∗∗*^	14.34 ± 0.44^a^^*∗∗*^	10.32 ± 0.33^a^^*∗∗*^
XVI	SFJE (400 mg/kg)	6.28 ± 0.27^a^^*∗∗*^	16.28 ± 0.86^a^^*∗∗*^	13.46 ± 0.34^a^^*∗∗*^	9.74 ± 0.11^a^^*∗∗*^
XVII	SFJE (600 mg/kg)	5.76 ± 0.13^a^^*∗∗*^	13.16 ± 0.55^a^^*∗∗*^	9.76 ± 0.30^a^^*∗∗*^	7.96 ± 0.19^a^^*∗∗*^
XVIII	SFJC (200 mg/kg)	6.52 ± 0.36^a^^*∗∗*^	16.88 ± 0.25^a^^*∗∗*^	14.78 ± 0.16^a^^*∗∗*^	10.58 ± 0.36^a^^*∗∗*^
XIX	SFJC (400 mg/kg)	5.78 ± 0.08^a^^*∗∗*^	15.24 ± 0.51^a^^*∗∗*^	12.8 ± 0.54^a^^*∗∗*^	9.74 ± 0.11^a^^*∗∗*^
XX	SFJC (600 mg/kg)	5.48 ± 0.28^a^^*∗∗*^	12.24 ± 0.42^a^^*∗∗*^	8.46 ± 0.42^a^^*∗∗*^	7.38 ± 0.44^a^^*∗∗*^

PFJM, methanolic extract of the fruit; PFJE, ethyl acetate extract of the fruit; PFJC, chloroform extract of the fruit; SFJM, methanolic extract of the seed; SFJE, ethyl acetate extract of the seed; SFJC, chloroform extract of the seed. When compared to the control group, the data values are shown as mean SEM (*n* = 5); ^a^^*∗*^*p* < 0.05 and ^a^^*∗∗*^*p* < 0.01 compared to the negative control group; ^b^^*∗*^*p* < 0.05 and ^b^^*∗∗*^*p* < 0.01 compared to the standard group (one-way ANOVA with post hoc Dunnett's test).

**Table 11:** Effect of *F. jangomas* extracts on castor oil-induced diarrhea in mice.

Group	Treatment	Total no. of feces in 4 h	% of inhibition
Negative control (I)	Tween 80 solution	13.4 ± 1.14	—
Standard (II)	Loperamide 2 mg/kg	1.6 ± 1.14^a^^*∗∗*^	88.06
III	PFJM (200 mg/kg)	10.4 ± 1.14^a^^*∗*^	22.39
IV	PFJM (400 mg/kg)	7.8 ± 0.84^a^^*∗∗*^^b^^*∗*^	41.79
V	PFJM (600 mg/kg)	6.4 ± 0.55^a^^*∗∗*^^b^^*∗∗*^	52.24
VI	PFJE (200 mg/kg)	11.4 ± 0.55^a^^*∗*^	14.93
VII	PFJE (400 mg/kg)	6.6 ± 0.89^a^^*∗*^	50.75
VIII	PFJE (600 mg/kg)	4.8 ± 0.84^a^^*∗∗*^	64.18
IX	PFJC (200 mg/kg)	10.2 ± 0.84^a^^*∗*^	23.88
X	PFJC (400 mg/kg)	6.6 ± 0.55^a^^*∗∗*^^b^^*∗*^	50.75
XI	PFJC (600 mg/kg)	4.2 ± 0.84^a^^*∗∗*^	68.66
XII	SFJM (200 mg/kg)	9.4 ± 0.55^a^^*∗*^	29.85
XIII	SFJM (400 mg/kg)	8 ± 0.71^a^^*∗*^	40.29
XIV	SFJM (600 mg/kg)	6.8 ± 0.45^a^^*∗*^	49.25
XV	SFJE (200 mg/kg)	9.2 ± 0.84^a^^*∗∗*^	31.34
XVI	SFJE (400 mg/kg)	7.4 ± 0.89^a^^*∗∗*^	44.78
XVII	SFJE (600 mg/kg)	4.6 ± 0.55^a^^*∗∗*^	65.67
XVIII	SFJC (200 mg/kg)	8 ± 0.71^a^^*∗∗*^	40.29
XIX	SFJC (400 mg/kg)	4.8 ± 0.84^a^^*∗∗*^	64.18
XX	SFJC (600 mg/kg)	4.4 ± 0.89^a^^*∗∗*^	67.16

PFJM, methanolic extract of the fruit; PFJE, ethyl acetate extract of the fruit; PFJC, chloroform extract of the fruit; SFJM, methanolic extract of the seed; SFJE, ethyl acetate extract of the seed; SFJC, chloroform extract of the seed. When compared to the control group, the data values are shown as mean SEM (*n* = 6); ^a^^*∗*^*p* < 0.05 and ^a^^*∗∗*^*p* < 0.01 compared to the negative control group; ^b^^*∗*^*p* < 0.05 and ^b^^*∗∗*^*p* < 0.01 compared to the standard group (one-way ANOVA with post hoc Dunnett's test).

**Table 12:** Effect of *F. jangomas* extracts on magnesium sulfate-induced diarrhea in mice.

Group	Treatment	Total no. of feces in 4 h	% of inhibition
Negative control (I)	Tween 80 solution	16 ± 1.23	—
Standard (II)	Loperamide 2 mg/kg	3.2 ± 1.30^a^^*∗∗*^	80.0
III	PFJM (200 mg/kg)	12.6 ± 0.89^a^^*∗*^	21.25
IV	PFJM (400 mg/kg)	9.8 ± 0.84^a^^*∗*^	38.75
V	PFJM (600 mg/kg)	7.6 ± 1.14^a^^*∗*^	52.5
VI	PFJE (200 mg/kg)	12.2 ± 0.84^a^^*∗*^	23.75
VII	PFJE (400 mg/kg)	9.6 ± 0.89^a^^*∗∗*^^b^^*∗*^	40.0
VIII	PFJE (600 mg/kg)	7.2 ± 0.45^a^^*∗∗*^^b^^*∗*^	55.0
IX	PFJC (200 mg/kg)	10.4 ± 0.55^a^^*∗∗*^	35.0
X	PFJC (400 mg/kg)	7.6 ± 0.55^a^^*∗∗*^^b^^*∗*^	52.5
XI	PFJC (600 mg/kg)	5.4 ± 0.55^a^^*∗∗*^^b^^*∗*^	66.25
XII	SFJM (200 mg/kg)	10.6 ± 0.55^a^^*∗*^	33.75
XIII	SFJM (400 mg/kg)	8.8 ± 0.84^a^^*∗*^	45.0
XIV	SFJM (600 mg/kg)	7.4 ± 0.85^a^^*∗∗*^^b^^*∗*^	53.75
XV	SFJE (200 mg/kg)	10.4 ± 0.86^a^^*∗*^	35.0
XVI	SFJE (400 mg/kg)	7.8 ± 0.87^a^^*∗*^	51.25
XVII	SFJE (600 mg/kg)	5.4 ± 0.88^a^^*∗∗*^^b^^*∗∗*^	66.25
XVIII	SFJC (200 mg/kg)	10.2 ± 0.89^a^^*∗∗*^	36.25
XIX	SFJC (400 mg/kg)	5.2 ± 0.90^a^^*∗∗*^	67.5
XX	SFJC (600 mg/kg)	4.2 ± 0.91^a^^*∗∗*^	73.75

PFJM, methanolic extract of the fruit; PFJE, ethyl acetate extract of the fruit; PFJC, chloroform extract of the fruit; SFJM, methanolic extract of the seed; SFJE, ethyl acetate extract of the seed; SFJC, chloroform extract of the seed. When compared to the control group, the data values are shown as mean SEM (*n* = 6); ^a^^*∗*^*p* < 0.05 and ^a^^*∗∗*^*p* < 0.01 compared to the negative control group; ^b^^*∗*^*p* < 0.05 and ^b^^*∗∗*^*p* < 0.01 compared to the standard group (one-way ANOVA with post hoc Dunnett's test).

**Table 13:** Effect of *F. jangomas* extracts on the gastrointestinal transit test in mice.

Group	Treatment	Total length of the intestine (cm)	Distance travel by charcoal (cm)	% of inhibition
Negative control (I)	Tween 80 solution	52.79 ± 0.17	53.14 ± 0.81	—
Standard (II)	Loperamide 2 mg/kg	54.78 ± 0.26	16.58 ± 0.33^a^^*∗∗*^	69.73
III	PFJM (200 mg/kg)	55.48 ± 0.59	30.60 ± 0.52^a^^*∗*^	44.84
IV	PFJM (400 mg/kg)	55.67 ± 0.68	27.33 ± 0.15^a^^*∗*^	50.91
V	PFJM (600 mg/kg)	56.62 ± 0.25	25.58 ± 0.24^a^^*∗*^	54.82
VI	PFJE (200 mg/kg)	55.56 ± 0.18	32.52 ± 0.40	41.47
VII	PFJE (400 mg/kg)	55.34 ± 0.95	28.51 ± 0.32^a^^*∗*^	48.48
VIII	PFJE (600 mg/kg)	55.29 ± 0.58	22.66 ± 0.18^a^^*∗*^	59.02
IX	PFJC (200 mg/kg)	58.64 ± 0.30	26.42 ± 0.08	54.94
X	PFJC (400 mg/kg)	57.88 ± 0.28	23.87 ± 0.1^a^^*∗*^	58.76
XI	PFJC (600 mg/kg)	56.76 ± 0.14	21.49 ± 0.17^a^^*∗∗*^	62.14
XII	SFJM (200 mg/kg)	54.92 ± 0.76	29.71 ± 0.46^a^^*∗*^	45.90
XIII	SFJM (400 mg/kg)	53.43 ± 0.21	23.43 ± 0.09^a^^*∗*^	56.15
XIV	SFJM (600 mg/kg)	53.65 ± 0.24	22.22 ± 0.01^a^^*∗*^	58.58
XV	SFJE (200 mg/kg)	54.64 ± 0.25	29.51 ± 0.21	45.99
XVI	SFJE (400 mg/kg)	52.87 ± 0.11	26.58 ± 0.27^a^^*∗*^	49.72
XVII	SFJE (600 mg/kg)	56.31 ± 0.05	24.62 ± 0.09^a^^*∗*^	56.28
XVIII	SFJC (200 mg/kg)	54.49 ± 0.28	27.61 ± 0.31^a^^*∗*^	49.33
XIX	SFJC (400 mg/kg)	55.09 ± 0.73	23.38 ± 0.25^a^^*∗*^	57.56
XX	SFJC (600 mg/kg)	55.27 ± 0.90	22.60 ± 0.40^a^^*∗*^	59.11

PFJM, methanolic extract of the fruit; PFJE, ethyl acetate extract of the fruit; PFJC, chloroform extract of the fruit; SFJM, methanolic extract of the seed; SFJE, ethyl acetate extract of the seed; SFJC, chloroform extract of the seed. When compared to the control group, the data values are shown as mean SEM (*n* = 6). ^a^^*∗*^*p* < 0.05 and ^a^^*∗∗*^*p* < 0.01 compared to the negative control group (one-way ANOVA with post hoc Dunnett's test).

## Data Availability

The data used to support the findings of this study are included within the article.
